# BAX as the mediator of C-MYC sensitizes acute lymphoblastic leukemia to TLR9 agonists

**DOI:** 10.1186/s12967-023-03969-z

**Published:** 2023-02-10

**Authors:** Ling Bai, Lei Zhou, Wei Han, Jingtao Chen, Xiaoyi Gu, Zheng Hu, Yongguang Yang, Wei Li, Xiaoying Zhang, Chao Niu, Yongchong Chen, Hui Li, Jiuwei Cui

**Affiliations:** 1 Cancer Center, The First Hospital of Jilin University, 1 Xinmin Street, Changchun, 130021 China; 2 Institute of Translational Medicine, The First Hospital of Jilin University, Changchun, 130021 China; 3 International Center of Future Science, Jilin University, Changchun, 130021 China

**Keywords:** Toll-like receptor 9, B-cell acute lymphoblastic leukemia, CpG oligodeoxynucleotide, Apoptosis, BAX

## Abstract

**Background:**

The prognosis of B-cell acute lymphoblastic leukemia (B-ALL) has improved significantly with current first-line therapy, although the recurrence of B-ALL is still a problem. Toll-like receptor 9 (TLR9) agonists have shown good safety and efficiency as immune adjuvants. Apart from their immune regulatory effect, the direct effect of TLR9 agonists on cancer cells with TLR9 expression cannot be ignored. However, the direct effect of TLR9 agonists on B-ALL remains unknown.

**Methods:**

We discussed the relationship between TLR9 expression and the clinical characteristics of B-ALL and explored whether CpG 685 exerts direct apoptotic effect on B-ALL without inhibiting normal B-cell function. By using western blot, co-immunoprecipitation, immunofluorescence co-localization, and chromatin immunoprecipitation, we explored the mechanism of the apoptosis-inducing effect of CpG 685 in treating B-ALL cells. By exploring the mechanism of CpG 685 on B-ALL, the predictive biomarkers of the efficacy of CpG 685 in treating B-ALL were explored. These efficiencies were also confirmed in mouse model as well as clinical samples.

**Results:**

High expression of TLR9 in B-ALL patients showed good prognosis. C-MYC-induced BAX activation was the key to the effect of CpG oligodeoxynucleotides against B-ALL. C-MYC overexpression promoted P53 stabilization, enhanced Bcl-2 associated X-protein (BAX) activation, and mediated transcription of the *BAX* gene. Moreover, combination therapy using CpG 685 and imatinib, a BCR-ABL kinase inhibitor, could reverse resistance to CpG 685 or imatinib alone by promoting BAX activation and overcoming BCR-ABL1-independent PI3K/AKT activation.

**Conclusion:**

TLR9 is not only a prognostic biomarker but also a potential target for B-ALL therapy. CpG 685 monotherapy might be applicable to Ph^−^ B-ALL patients with C-MYC overexpression and without BAX deletion. CpG 685 may also serve as an effective combinational therapy against Ph^+^ B-ALL.

**Supplementary Information:**

The online version contains supplementary material available at 10.1186/s12967-023-03969-z.

## Key points

- TLR9 is not only a prognostic biomarker but also a potential target for B-ALL therapy.
- C-MYC-mediated BAX activation leads to CpG 685-induced apoptosis in B-ALL.

**Supplementary Information:**

The online version contains supplementary material available at 10.1186/s12967-023-03969-z.

## Introduction

Acute lymphoblastic leukemia (ALL) is a malignant disease characterized by abnormal transformation and proliferation of immature lymphocytes, and approximately 75% of people with this disease have a precursor B-cell immunophenotype [1]. More than 90% of patients with B-cell ALL (B-ALL) achieve complete remission after chemotherapy, but as many as half of the patients often relapse due to the presence of measurable residual disease (MRD) [2].

Immunotherapy, as an effective therapy for the elimination of MRD, can significantly reduce the recurrence of B-ALL [3, 4]. Indeed, owing to the low immunogenicity of B-ALL cells, the effect of current immunotherapy is still limited [5]. Even though T cells with chimeric antigen receptors have improved the response rate of B-ALL from 30 to 90% [6], toxicity also limits its use [7, 8]. Thus, it is critical to find novel strategies for B-ALL treatment to reduce side effects and prolong survival.

Toll-like receptor 9 (TLR9), a natural immunoregulatory site, bridges innate and adaptive immunity. Apart from their immune regulatory effect, the direct effect of TLR9 agonists on cancer cells with TLR9 expression cannot be ignored [9]. In addition, as B-cell malignancies arise from well-differentiated B cell subtype, they can be recognized by TLR9 agonists just like B cells [10]. However, B-type CpG oligodeoxynucleotides (CpG ODNs), a subtype of TLR9 agonists, induce proliferation, plasma cell differentiation, and immunoglobulin secretion in normal B cells [11], whereas their effects on B-cell malignancies are disease-specific, including proliferation, apoptosis, or anergy. CpG ODNs also have opposite direct effects on different subtypes of the same cancer. [11]

Our present study aimed to clarify the expression of TLR9 and the direct effect of CpG ODNs on B-ALL cells to determine whether CpG ODNs can be used as a novel drug that can kill B-ALL cells while improving their immunogenicity. We also wanted to explore the mechanism and combination therapeutic strategies of CpG ODNs against B-ALL with different characteristics, such as Philadelphia (Ph) chromosome positive (Ph^+^) and Ph chromosome negative (Ph^−^) [12] B-ALL cells, which may increase our understanding of the effectiveness of CpG ODNs. It was possible to find out the reasons for the individual differences in the sensitivity of CpG ODNs in treatment, and optimize the precise treatment of B-ALL. Moreover, our study might propose TLR9 as a new therapeutic target for reducing recurrence and improving the overall survival of B-ALL patients.

## Results

### TLR9 is widely expressed in B-ALL cells and reflects better prognosis

**Fig. 1:**
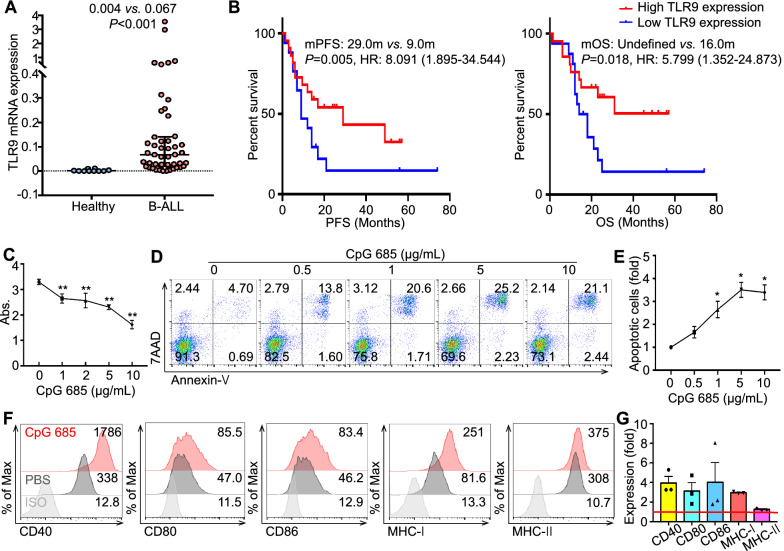
TLR9 is highly expressed in B-ALL cells and an independent prognostic factor for B-ALL **A**. Real-time qRT-PCR results of the TLR9 expression profile in PBMC from 49 B-ALL patients and 10 healthy controls and normalized to the expression of *β-actin*. The expression level of TLR9 in the PBMC of B-ALL patients was significantly higher than that of healthy people (Medium: 0.004 *vs*. 0.067). All data are shown with medium and interquartile range. Significant difference was *p* < 0.001. **B** Cox multivariate model indicated that the expression level of *TLR9* is an independent prognostic factor of B-ALL, which is related to PFS (mPFS: 29.0 months *vs*. 9.0 months, *p* = 0.005, HR: 8.091) and OS (mOS: undefined *vs*. 16.0 months, *p* = 0.018, HR: 5.799). And B-ALL patients with high *TLR9* expression have a better prognosis. **C** CpG 685 inhibits proliferation in BLIN-1 cells. WST-1 cell viability assay results showing kinetic changes in BLIN-1 cell number after 3 day culture in media with different doses of CpG 685. Columns represent means of 5 independent experiments; bars represent SD. **p < 0.01. **D** CpG 685 induces apoptosis in BLIN-1 cells. Viable BLIN-1 cells cultured in media with different doses of CpG 685 after 3-day culture were examined by annexin-V/7AAD staining. The percentage of apoptotic BLIN-1 cells was calculated. CpG 685 induces BLIN-1 cells to apoptosis in a CpG 685 dose-dependent manner.** E** The percentage of Annexin-V^+^ cells in BLIN-1 cells (fold) with different doses of CpG 685 after 3 day culture was analyzed by flow cytometry. Columns represent means of 3 independent experiments; bars represent SEM. *p < 0.05. **F** CpG 685 upregulates CD40, CD80, CD86, MHC-I, and MHC-II expression, further promoting the antigen presentation function of BLIN-1 cells. **G** The folds of costimulatory molecules on the surface of BLIN-1 cells were upregulated after 24 h of CpG 685 stimulation compared with those without CpG 685. The red line represents no upregulation. Columns represent means of 3 independent experiments; bars represent SEM

Data from the Human Protein Atlas (https://www.proteinatlas.org/) showed that TLR9 expression varied in different B-cell subtypes (Additional file 1: Fig. S1A). B-ALL patients (N = 49) in our hospital showed higher TLR9 expression in peripheral blood mononuclear cells (PBMCs) than healthy controls (N = 10) (median: 0.004 *vs.* 0.067, *p* < 0.001, Fig. 1A); thus, it is indicated that TLR9 is widely expressed in B-ALL cells.

Studies have shown that based on the function of TLRs in the immune system, the expression of TLRs on cancer cells was correlated with the prognosis of cancer patients [13, 14]. However, the results were inconsistent. To clarify whether TLR9 is an independent prognostic factor of B-ALL, we divided B-ALL patients into TLR9 high expression group (N = 23) and low expression group (N = 18) based on the median expression of TLR9. Taking the relevant factors affecting the baseline balance (Additional file 1: Table S3) and the factors that may affect prognosis in the univariate analysis as confounding factors into the Cox multivariate model (*p* < 0.1, Additional file 1: Table S4), the expression level of TLR9 was found to be an independent prognostic factor of B-ALL (Fig. 1B), which is related to progression free survival (PFS) (mPFS: 29.0 months *vs.* 9.0 months, *p* = 0.005, HR: 8.091) and overall survival (OS) (mOS: undefined *vs.* 16.0 months, *p* = 0.018, HR: 5.799). It is indicated that B-ALL patients with high TLR9 expression have better prognosis.

### CpG 685 suppresses the proliferation, induces apoptosis, and upregulates costimulatory molecules of BLIN-1 cells

To explore the anti-B-ALL effect of TLR9 agonists, we selected four B-ALL cell lines (BLIN-1, RS4;11, NALM-6, and Sup-B15) as models and verified their TLR9 expression according to RT-qPCR (Additional file 1: Fig. S1C). Both B-type and C-type CpG ODNs induced apoptosis in BLIN-1 and RS4;11 cells. Moreover, CpG 685 showed a stronger effect on B-ALL than other TLR9 agonists tested (Additional file 1: Figure S1D).

To clarify the direct effect of CpG 685 on B-ALL cells, we examined the effect of CpG 685 on BLIN-1. BLIN-1 cells cultured in media grew rapidly, and a WST-1 cell viability assay showed that CpG 685 inhibited their growth. After 3 days of culture, approximately 50% BLIN-1 cells were inhibited by 10 µg/mL CpG 685 relative to the inhibition of untreated cells (Fig. 1C). Besides, kinetic analysis showed significant decreases in viable Annexin-V/7-AAD double-negative BLIN-1 cells after 3 days of culture with CpG 685 (Fig. 1D, E). This inhibitory effect was dependent on CpG 685 concentration and treatment time (Additional file 1: Figure S2A). Moreover, CpG 685 treatment upregulated the expression of costimulatory molecules, especially CD40, CD80, and MHC-I (Fig. 1F, G), and other immunoregulatory molecules (Fas, FasL, DR4, DR5, and MICA) (Additional file 1: Figure S2C), increasing the recognition and killing of BLIN-1 cells by the immune system.

### CpG 685-induced apoptosis of BLIN-1 cells activates TLR9 downstream signaling pathways

**Fig. 2:**
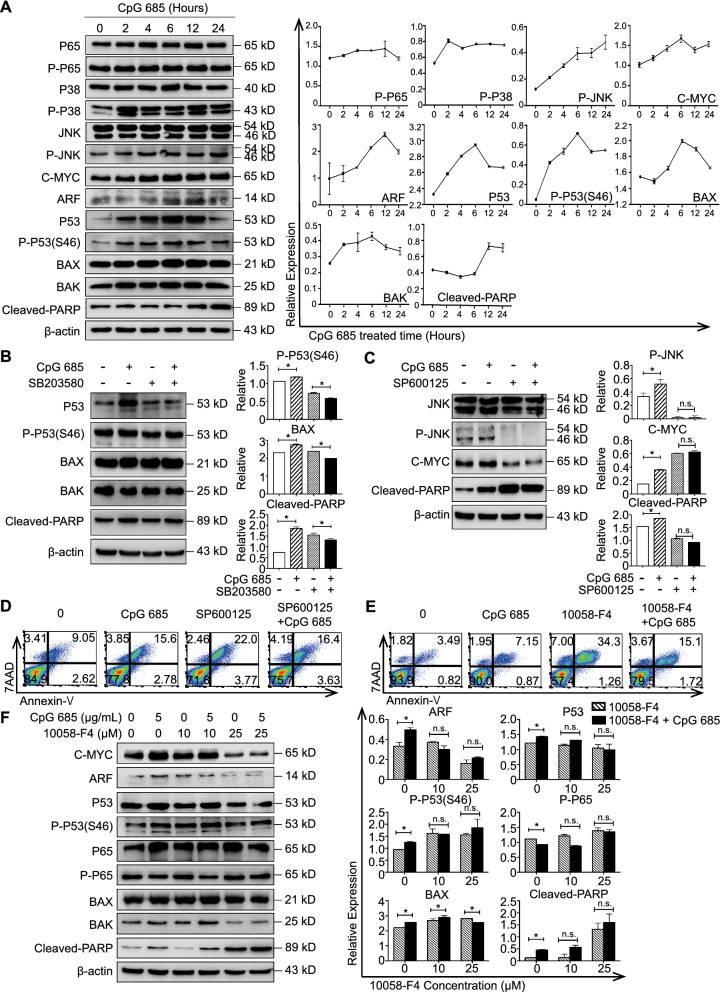
CpG 685-induced apoptosis of BLIN-1 cells is P38/P53/BAX and JNK/C-MYC signaling pathway dependent. **A** CpG 685 activates downstream molecules of the TLR9 signaling pathway in BLIN-1 cells. Phosphorylated P65, phosphorylated P38, phosphorylated JNK, C-MYC, ARF, P53, phosphorylated p53 at ser46, BAX, BAK, and PARP cleavage in BLIN-1 cells cultured with or without 5 μg/mL CpG 685 were examined by western blotting at the indicated time points (left panel). All data shown are representative of 3 independent experiments (right panel), mean ± SD. **B** BLIN-1 cells with or without SB203580 pretreatment were cultured in media with or without CpG 685 for 24 h. Phosphorylated p53 at ser46, BAX expression, and PARP cleavage were reversed by SB203580 pretreatment of BLIN-1 cells under CpG 685 treatment (left panel). All data shown are representative of 3 independent experiments (right panel), mean ± SD. *p < 0.05. **C** BLIN-1 cells with or without SP600125 pretreatment were cultured in media with or without CpG 685 for 24 h. The activations of C-MYC and PARP cleavage of BLIN-1 cells under CpG 685 treatment can be reversed by SP600125 pretreatment (left panel). All data shown are representative of 3 independent experiments (right panel), mean ± SD. *p < 0.05. **D** JNK inhibition by SP600125 enabled BLIN-1 to overcome CpG 685 induced apoptosis. Cells were incubated alone or in the presence of CpG 685 with or without 40 μM SP600125 pretreatment for 72 h. Cells were then harvested and labeled with antibodies against Annexin-V and 7AAD to define the apoptotic cells. **E** C-MYC inhibition by 10058-F4 enabled BLIN-1 to overcome CpG 685 induced apoptosis. Cells were incubated alone or in the presence of CpG 685 with or without 25 μM 10058-F4 pretreatment for 72 h. Cells were then harvested and labeled with antibodies against Annexin-V and 7AAD to define the apoptotic cells. **F** BLIN-1 cells with different doses of 10058-F4 pretreatment were cultured in media with or without CpG 685 for 24 h. ARF expression, P53 expression, activation of phosphorylated p53 at ser46, BAX expression, and PARP cleavage were reversed by 25 μM 10058-F4 pretreatment before CpG 685 stimulation (left panel). Densitometry of western blots was analyzed with ImageJ and is presented as a mean ± SD at each dose point on the right panel. Columns represent means of at least 3 independent experiments. Significant differences were accepted at *p < 0.05 against the group with no CpG 685 at each dose of inhibitor pretreatment and calculated according to the two-tailed Student’s *t*-test

The P38 MAPK, JNK, and NF-κB signaling pathways are downstream of TLR9 in normal B cells, whereas the ERK pathway is not activated by CpG ODNs. [15] To understand the mechanisms of TLR9-induced apoptosis in B-ALL, we examined key molecules of these pathways via western blotting. We found that P38 phosphorylation increased in the first 2 h, and P53 phosphorylation on Ser46 showed a similar trend (Fig. 2A).

Both JNK and C-MYC have dual effects. JNK has three subtypes, including JNK1 (46 kDa) and JNK2 (54 kDa) that are expressed in cancer cells. JNK1 activation may induce apoptosis, whereas JNK2 activation inhibits it [16]. Our results showed the sustained activation of JNK1 (Fig. 2A).

C-MYC is a potential downstream molecule of JNK1-mediated apoptosis [17], and the apoptotic role of C-MYC depends on C-MYC overexpression and apoptotic pathway activation [18]. Our results revealed that ARF and P53 expression was upregulated following the upregulation of C-MYC. With the increase in C-MYC, a significant change in BAX protein levels was observed as early as 4 h after stimulation, with sustained elevation for at least 20 h. Poly ADP-ribose polymerase (PARP) cleavage began 12 h after CpG 685 stimulation, suggesting that apoptosis began at this time. (Fig. 2A).

### CpG 685-induced apoptosis of BLIN-1 cells is dependent on the P38 MAPK and JNK/C-MYC signaling pathways

To investigate the TLR9 downstream pathway of P38 MAPK, we used the P38 MAPK-specific inhibitor SB203580 to inhibit the activation of P38 substrates [19]. Results showed that P53 phosphorylation at Ser46, BAX expression, and PARP cleavage were abrogated by 40 μM SB203580 after CpG 685 treatment (Fig. 2B). These results suggested that P38 plays a pro-apoptotic role, inducing phosphorylation of the tumor suppressor protein P53 on Ser46, thus promoting BAX transcription and leading to cell apoptosis.

JNK cooperates with other signal transduction pathways to mediate cell survival. Sustained JNK activation can induce apoptosis. [20, 21] To detect the apoptotic roles of JNK signaling pathways in BLIN-1 cells, we used the specific JNK inhibitor SP600125 to block JNK phosphorylation and its downstream signaling [22]. The results showed that 40 μM SP600125 blocks the increase in C-MYC expression and PARP cleavage induced by CpG 685 for 24 h (Fig. 2C). Flow cytometry also indicated that SP600125 pretreatment inhibited CpG 685-induced apoptosis in BLIN-1 cells (Fig. 2D, Additional file 1: Figure S2E). This confirms that CpG 685-induced apoptosis depends on the JNK/C-MYC pathway.

### C-MYC-mediated BAX activation leads to CpG 685-induced caspase-independent apoptosis

Although the P38 MAPK and JNK/C-MYC pathways played apoptotic roles in the stimulation of BLIN-1 cells with CpG 685, how this C-MYC-mediated pathway relates to apoptosis is unclear. To verify the importance of C-MYC and its regulated downstream molecules, we used the C-MYC inhibitor 10,058-F4 to prevent the expression of *C-MYC* target genes and to inhibit C-MYC-MAX interactions [23]. WST-1 assay showed that after 24 h, 25 μM 10,058-F4 pretreatment inhibited the decrease in BLIN-1 cell number due to CpG 685 (Additional file 1: Figure S2D). Flow cytometry also indicated that 10,058-F4 pretreatment inhibited CpG 685-induced apoptosis in BLIN-1 cells (Fig. 2E, Additional file 1: Figure S2E). Thus, C-MYC expression is the key to CpG 685-mediated apoptosis.

To confirm the downstream signaling pathways of C-MYC apoptotic effect, we used 10,058-F4 pretreatment or C-MYC siRNA transfection to inhibit the expression of C-MYC as well as its possible downstream target genes. Our results revealed that ARF expression, P53 expression, BAX expression, BAK expression, and PARP cleavage were upregulated by CpG 685 stimulation. Nevertheless, 25 μM 10058-F4 pretreatment could block the CpG 685-induced increase in these proteins, except for BAK (Fig. 2F). Interfering with the expression of C-MYC also could block the cleavage of PARP, which was induced by CpG 685 (Additional file 1: Fig. 2F). Thus, these results indicate that ARF, P53, and BAX are the downstream molecules of C-MYC. C-MYC can promote B-ALL apoptosis by promoting the cleavage of PARP in the downstream signaling pathway of TLR9.

**Fig. 3:**
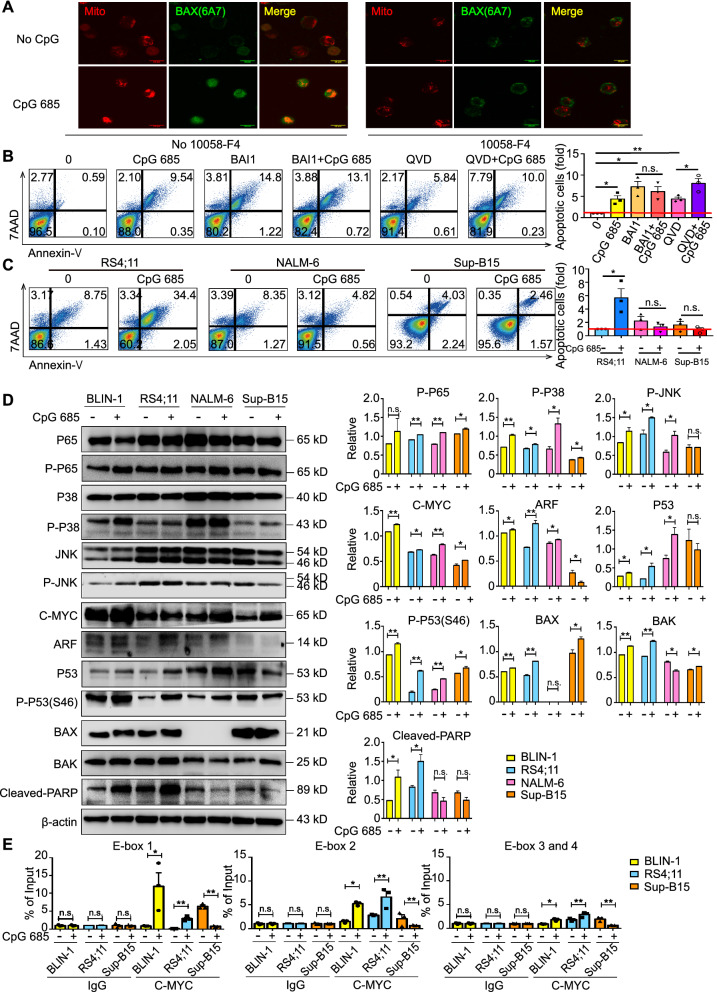
C-MYC-mediated BAX activation leads to CpG 685-induced caspase-independent apoptosis. **A** BLIN-1 cells with or without 25 μM 10058-F4 pretreatment were cultured in media with or without CpG 685 for 24 h. The expression and localization of Mito Tracker (red) and BAX(6A7) (green) were detected and evaluated by immunofluorescence and laser scanning confocal microscopy. CpG 685 can promote the expression of BAX(6A7) when C-MYC is activated and participate in the enrichment of BAX(6A7) to mitochondria. That is, 10058-F4 can block the activation of BAX by CpG 685. All images are analyzed by ImageJ software. **B** BAI1 is an inhibitor of BAX conformational activation. QVD represents Quinoline-Val-Asp-Difluorophenoxymethylketone (Q-VD-OPh) is a kind of effective pan-caspase inhibitor, which can prevent the cleavage of PARP mediated by caspase cascade. Apoptotic and viable BLIN-1 cells were determined by Annexin V/7AAD staining. Kinetic analysis showed that 0.5 μM BAI1 pretreatment inhibited CpG 685 induced Annexin V-positive apoptotic BLIN-1 cell number, while 80 μM QVD pretreatment did not block CpG 685 induced BLIN-1 apoptosis (left panel). The percentage of Annexin-V^+^ cells (fold) was analyzed by flow cytometry (right panel). Columns represent means of 3 independent experiments; bars represent SEM. *p < 0.05, **p < 0.01. The red line represents onefold. **C** Viable B-ALL cells cultured in media with or without 5 μg/mL CpG 685 after 3 day. Cells were examined by annexin-V/7AAD staining. The percentage of annexin V-positive B-ALL cells represent the apoptotic cells. CpG 685 induces apoptosis in RS4;11 cells, while NALM-6 and Sup-B15 cells are resistant to CpG 685 treatment (left panel). The percentage of Annexin-V^+^ cells (fold) was analyzed by flow cytometry (right panel). Columns represent means of 3 independent experiments; bars represent SEM. *p < 0.05. The red line represents onefold. **D** By comparing the protein expression levels of B-ALL cells with or without CpG 685, western blot results showed that three Ph( − ) B-ALL cells (BLIN-1, RS4;11, and NALM-6) overexpressed C-MYC compared to levels in Sup-B15 cells. Although CpG 685 can activate P38/P53 signaling pathway in BLIN-1, RS4;11, and NALM-6 cells, it cannot induce apoptosis in NALM-6 due to the loss of BAX expression. Besides, CpG 685 also cannot promote PARP cleavage in Ph( +) B-ALL cell (Sup-B15). Columns in the right panel represent means of at least 3 independent experiments; bars represent SD. Significant differences were accepted at *p < 0.05 against the group with no CpG 685 on each cell line. **p < 0.01. **E** With IgG as the control and input as the benchmark, ChIP-qPCR was used to determine the binding of C-MYC to the E-box region of the *bax* promoter. BLIN-1 and RS4;11 cells stimulated by CpG 685 for 12 h can promote the combination of C-MYC and E-box 1, 2, 3 and 4 C-MYC could not directly bind to the BAX promoter on Sup-B15 cells. All data are shown with mean ± SEM. Significant differences were accepted at *p < 0.05. **p < 0.01

To further clarify the direct regulation of C-MYC on apoptotic signaling pathways, ChIP qPCR results showed that BLIN-1 cells stimulated by CpG 685 for 12 h can promote the direct binding of C-MYC to E-box 1, 2, 3, and 4 of the *BAX* promoter (Fig. 3E). Moreover, CpG 685 promoted the expression of BAX (6A7), suggesting BAX activation (Fig. 3A). Furthermore, activated C-MYC integrated BAX (6A7) into the outer mitochondrial membranes following treatment with CpG 685 for 24 h (Fig. 3A); thus, C-MYC participates in the transcription and activation of BAX in BLIN-1 cells following CpG 685 stimulation.

Moreover, although previous studies have shown that C-MYC mediated apoptosis signaling through the mitochondrial pathway[24], whether it is dependent on BAX or caspase has not been reported [25–28]. Thus, we used the BAX conformational activation inhibitor BAI1 and the pan-caspase inhibitor Q-VD-OPh to block different stages of mitochondrial apoptosis pathway. Our results revealed that CpG 685-induced caspase-independent apoptosis in BLIN-1 relied on BAX activation (Fig. 3B).

### CpG 685 promotes apoptosis in Ph^−^ B-ALL cells with C-MYC overexpression and without BAX deletion

Above all, CpG 685 activated the P38/P53/BAX and JNK/C-MYC pathways in B-ALL cells. C-MYC expression on B-ALL cells promoted BAX transcription and activation as well as ARF expression, which further caused caspase-independent apoptosis in B-ALL cells under CpG 685 stimulation. To determine the general applicability of the above mechanism in B-ALL cells, we repeated the experiment in other three B-ALL cell lines (RS4;11, NALM-6, and Sup-B15).

Results showed that CpG 685 also inhibited the proliferation of RS4;11, but not that of NALM-6 and Sup-B15 (Additional file 1: Figure S2G). Similarly, CpG 685 induced apoptosis in RS4;11, but not in NALM-6 and Sup-B15 (Fig. 3C).

To investigate the mechanisms of the heterogeneous effects of CpG 685 on B-ALL cells, we compared the downstream molecules mediated by CpG 685 in different cell lines (Fig. 3D; Additional file 1: Figure S2H, I). Results revealed that CpG 685 could not induce apoptosis in NALM-6 because of BAX deletion (Fig. 3D; Additional file 1: Figure S2I). Moreover, not only Sup-B15 has low C-MYC expression compared with the other cell lines, it is also a Ph^+^ B-ALL cell line. BCR-ABL upregulates many signaling pathways, including JAK/STAT, C-MYC, and PI3K/AKT/mTOR. Multiple downstream pathways of BCR-ABL could inhibit BAX activation [29, 30] and further suppressed CpG 685-induced apoptosis in Ph^+^ B-ALL cells (Fig. 3D). In addition, in Sup-B15, C-MYC not only could not promote ARF and P53 expression but also could not directly bind to the *BAX* promoter after CpG 685 stimulation, suggesting that C-MYC regulates different target genes in Sup-B15 than in BLIN-1 and RS4;11 (Fig. 3E, Additional file 1: Figure S2B).

AIF is the most important gene product related to caspase-independent apoptosis [31]. BAX activation can promote the translocation of AIF from the mitochondria to the nucleus and induce chromatin condensation [32]. By detecting the expression and location of AIF in both BLIN-1 and RS4;11 after CpG 685 treatment, we confirmed that CpG 685-induced apoptosis of B-ALL was caspase-independent (Additional file 1: Figure S2J).

### Different C-MYC target genes lead to the resistance of Sup-B15 cells to CpG 685

To further clarify the difference in Ph^+^ B-ALL and Ph^−^ B-ALL reactivity to CpG 685, we compared the effects and the downstream molecules in different time periods mediated by CpG 685 in Sup-B15. No proliferation suppression (Additional file 1: Figure S3A), apoptosis (Additional file 1: Figure S3B), or increased antigen presentation (Additional file 1: l Figure S3C) was observed, and only FasL and DR5 were slightly upregulated (Additional file 1: Figure S3D) in Sup-B15 cells after CpG 685 stimulation.

**Fig. 4:**
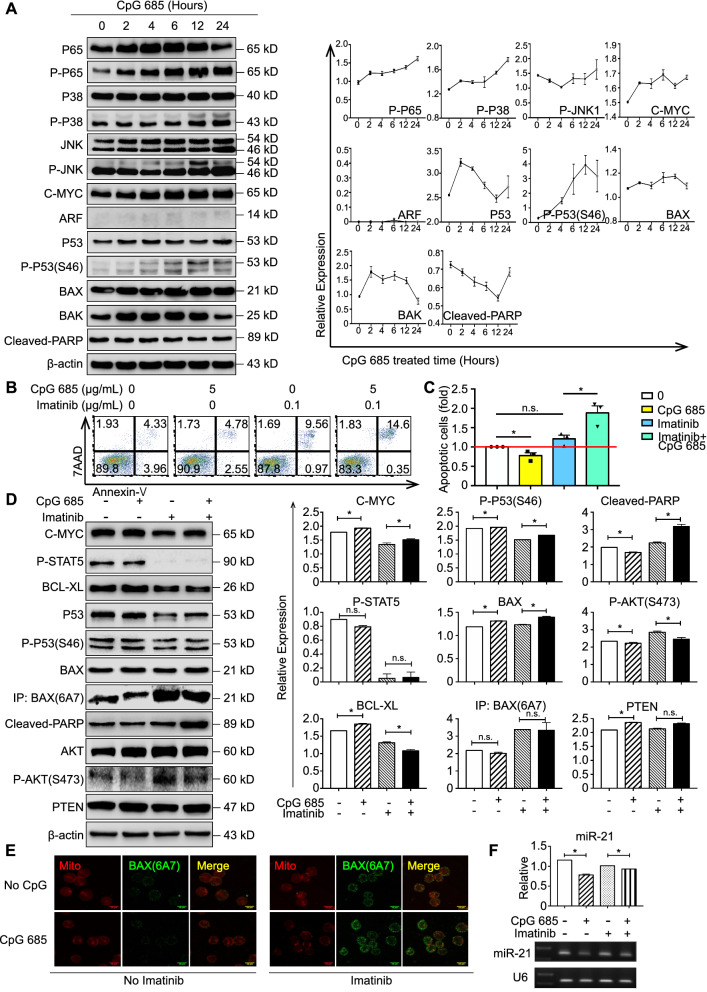
Sup-B15 cells are resistant to CpG 685 treatment and imatinib reverses the resistance **A**. The expression of phosphorylated P65, phosphorylated P38, C-MYC, phosphorylated p53 at ser46, and BAX have increased by 5 μg/mL CpG 685 treatment at the indicated time points in Sup-B15 cells, as determined by western blotting (left panel). However, the expression of JNK1 phosphorylation is decreased by CpG 685 treatment and ARF is almost undetectable. Densitometry of western blots was analyzed with ImageJ and is presented as a mean ± SD at each time point on the right panel. **B** Annexin-V/7AAD staining of apoptotic Sup-B15 cells with or without 0.1 mg/L imatinib pretreatment after culture in media with or without CpG 685 for 3 days. Combinational use of CpG 685 and imatinib to treat Sup-B15 cells reversed resistance to CpG 685 treatment alone. **C** The percentage of Annexin-V^+^ cells in Sup-B15 cells (fold) which were incubated alone or in the presence of CpG 685 with or without imatinib pretreatment for 3-day was analyzed by flow cytometry. Columns represent means of 3 independent experiments; bars represent SEM. *p < 0.05. **D** Western blot results showing that imatinib inhibits downstream molecules of BCR-ABL, such as phosphorylated STAT5, C-MYC, and BCL-XL. Active BAX was upregulated by imatinib and eventually induced apoptosis of Sup-B15 cells with CpG 685. Imatinib upregulates the phosphorylation of AKT at ser473. At the same time, CpG 685 dephosphorylates AKT at ser473 by upregulating PTEN, and ultimately reversed the drug resistance of imatinib, which caused by BCR-ABL1-independent AKT phosphorylation in Ph( +) B-ALL. (left panel) Results are presented as the mean ± SD on the right panel. *p < 0.05. **E** Immunofluorescence and laser scanning confocal microscopy showed that imatinib promotes the expression of BAX(6A7). BAX(6A7) staining represented the activated BAX. The combination therapy of CpG 685 and imatinib is a benefit for the BAX activation. **F** RT-PCR (down penal) and qRT-PCR (up penal) results of miR-21 and U6. MiR-21 was quantitated by normalizing ΔΔCt value over U6. CpG 685 downregulated miR-21 with or without imatinib in Sup-B15 cells. QRT-PCR results is presented as a mean ± SD at each time point on the right panel. Significant difference was accepted at *p < 0.05

Despite the fact that P38 activation promoted P53^ser46^ phosphorylation and BAX expression (Additional file 1: Figure S3E), the upregulation of JNK1 phosphorylation, ARF expression, or PARP cleavage did not occur in Sup-B15 cells after CpG 685 stimulation (Fig. 4A). Moreover, CpG 685 could not activate BAX in Sup-B15 (Additional file 1: Figure S3F). Thus, C-MYC could not promote ARF expression, BAX activation, and apoptosis in Sup-B15 cells after CpG 685 treatment.

### Combination of CpG 685 and imatinib reverses Sup-B15 resistance to CpG 685 or imatinib alone

To determine whether BCR-ABL was the key factor of Ph^+^ B-ALL resistance to CpG 685, we used imatinib to inhibit its kinase activity. The results showed that imatinib combined with CpG 685 significantly increased Annexin-V/7-AAD-positive apoptotic cells among Sup-B15 cells compared with CpG 685 alone (Fig. 4B, C).

To further clarify the mechanism by which imatinib reverses CpG 685 resistance in Sup-B15, we detected the downstream molecules of BCR-ABL. Results showed that phosphorylated STAT5, C-MYC, and BCL-XL were significantly decreased by imatinib (Fig. 4D). Due to BCL-XL could block BAX translocation [33], imatinib decreased BCL-XL expression and further reactivated BAX (6A7) expression, leading to apoptosis in Ph^+^ B-ALL cells (Fig. 4D). Immunofluorescence analysis also confirmed that imatinib promoted the expression of BAX (6A7) (Fig. 4E). Furthermore, P53 phosphorylation and BAX expression were not suppressed by imatinib pretreatment, further confirming the antiapoptotic effect of C-MYC in Sup-B15, different from that in Ph^−^ B-ALL cells (Fig. 4D). Thus, imatinib combined with CpG 685 not only blocked the bypassing pathways that affect BAX activation but also inhibited the different target genes of C-MYC in Sup-B15, which exerted antiapoptotic effects.

Interestingly, Sup-B15 cells are imatinib-resistant, and the resistance mechanism is related to PI3K/AKT/mTOR pathway overactivation after treatment [34]. Targeting miR-21 can upregulate PTEN expression, which sensitizes Sup-B15 cells to imatinib-induced apoptosis [35]. Our results revealed that combined use of CpG 685 and imatinib also reversed the resistance to imatinib monotherapy (Fig. 4B, C Additional file 1: Figure S4A). CpG 685 downregulated miR-21, further upregulated PTEN, and ultimately reversed the drug resistance of imatinib caused by BCR-ABL1-independent AKT phosphorylation at Ser473 in Ph^+^ B-ALL cells (Fig. 4D, F, Additional file 1: Figure S4B).

### CpG 685 treatment reduces the B-ALL burden and prolongs survival of B-ALL-engrafted NCG mice

**Fig. 5:**
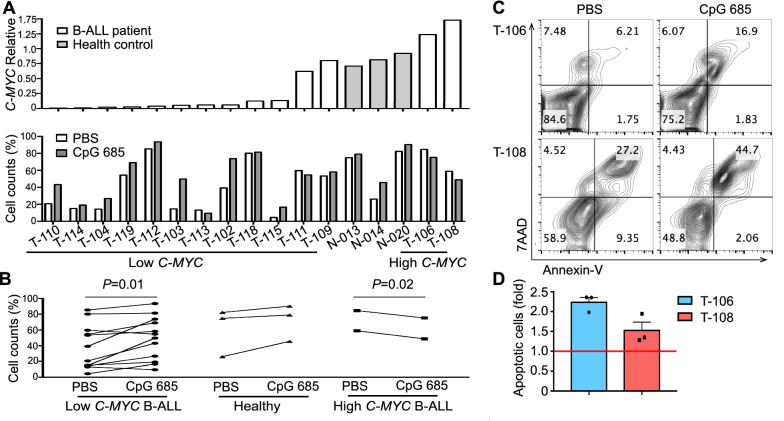
CpG 685 promotes the apoptosis in primary Ph^−^ B-ALL cells with C-MYC overexpression and without BAX deletion. **A** Real-time RT-PCR results of *c-myc* expression profile in PBMC from 14 B-ALL patients and 3 healthy controls and normalized to the expression of *β-actin* (up penal). Viable B-ALL cell number in B-ALL cells from these patients cultured in media with or without 5 μg/ml CpG 685 at day 3 of culture were determined by annexin-V/7AAD staining. Viable B-ALL cell number was calculated and ordered by the levels of *c-myc* expression (down penal). **B** Using the *c-myc* expression level in PBMC of healthy control as the reference, B-ALL patients were divided into *c-myc* high expression group and *c-myc* low expression group. Results found that CpG 685 can reduce the viable B-ALL cells in the *c-myc* high expression group according to the two-tailed paired *t*-test. (*p* = 0.02). **C** Annexin-V/7AAD staining of B-ALL cells in patients with high expression of *c-myc*, CpG 685 can significantly promote the apoptosis of B-ALL cells. **D** The percentage of Annexin-V^+^ B-ALL cells (fold) from 2 patients with high expression of *c-myc* treated with or without CpG 685 after 3 day was analyzed by flow cytometry. Columns represent means of 3 independent experiments; bars represent SEM

Apart from cell line experiment, we also tested the effect of CpG 685 or PBS on primary B-ALL cells without BAX deletion in vitro (Additional file 1: Table S5). Using the median of *C-MYC* expression level in the PBMCs of healthy controls as a reference, B-ALL patients were divided into *C-MYC* high expression group and *C-MYC* low expression group. Results showed that CpG 685 reduced the number of viable Ph^−^ B-ALL cells in the *C-MYC* high expression group, according to two-tailed paired* t*-test (*p* = 0.02, Fig. 5A, B). It is also indicated that CpG 685 might significantly promoted apoptosis in primary Ph^−^ B-ALL cells with C-MYC overexpression (Fig. 5C, D).

**Fig. 6:**
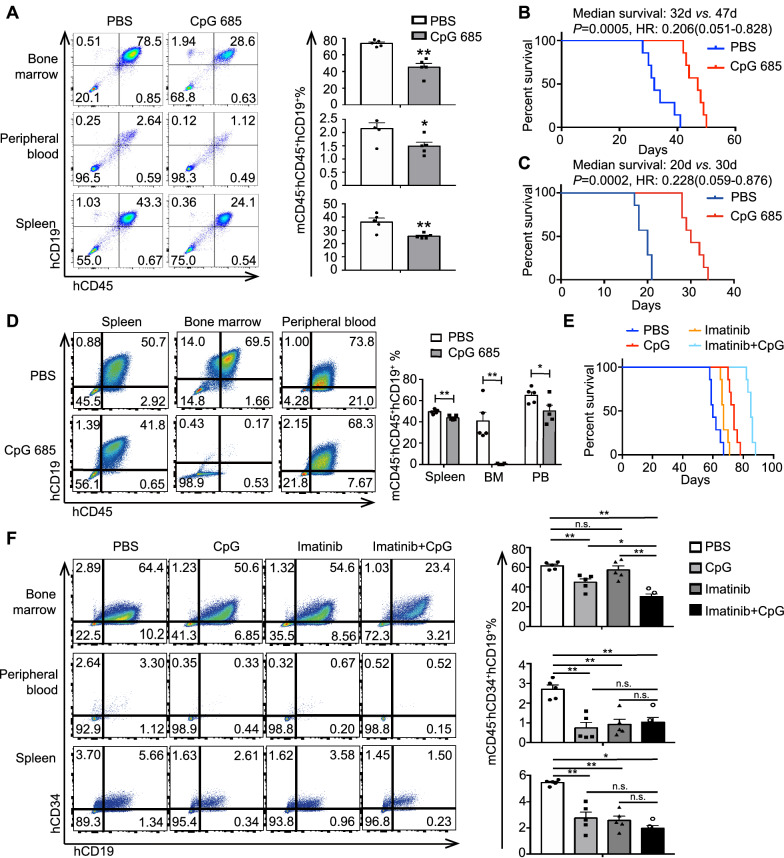
CpG 685 treatment reduces B-ALL burden systemically in vivo. **A** CpG 685 treatment reduces BLIN-1 cell engraftment in NCG mouse xenograft models. Cohorts of mice were i.v. injected with 5 × 10^6^ BLIN-1 cells, which led to the development of a lethal leukemia that killed all BLIN-1 mice within 6 weeks. After 15 days, cohorts of mice were also i.p. injected with 2 mg/kg CpG 685 or PBS control for the next 5 days. On day 30, flow cytometry was used to detect the percentage of hCD19^+^hCD45^+^/mCD45^+^ cells in peripheral blood, spleen and bone marrow. The percentage of hCD19^+^hCD45^+^/mCD45^+^ cells represent BLIN-1 cells infiltrating in peripheral blood, spleen and bone marrow. The infiltration degree of BLIN-1 cells in bone marrow, spleen and peripheral blood of mice was different, mainly in bone marrow, followed by spleen. Compared with PBS, CpG 685 could significantly inhibit the infiltration of BLIN-1 cells in bone marrow, spleen and peripheral blood, especially the infiltration of BLIN-1 cells in bone marrow. (left panel) Aggregated results detected from 5 BLIN-1 mice of each group and are presented as the mean ± SD (right panel). *p < 0.05, **p < 0.01. **B** Survival curve of BLIN-1 mice of each group (7 mice per group). CpG 685 significantly prolonged the survival of BLIN-1 mice compared with PBS (median survival: 32 days *vs.* 47 days, HR: 0.206). *p* = 0.0005. **C** Survival curve of B-ALL PDX mice of each group (7 mice per group). CpG 685 significantly prolonged the survival of B-ALL PDX mice compared with PBS (median survival: 20 days *vs.* 30 days, HR: 0.228). *P* = 0.0002. **D** CpG 685 treatment on B-ALL PDX model. Cohorts of mice were i.v. injected with 1 × 10^6^ B-ALL cells. At days 10, blood is taken through the tail vein to check the tumor burden. According to the proportion of hCD19^+^hCD45^+^/mCD45^+^ cells in peripheral blood, PDX model mice were divided into two groups. Each cohort of mice were also i.p. injected with 2 mg/kg CpG 685 or PBS control for the next 5 days. On day 22, flow cytometry was used to detect the percentage of hCD19^+^hCD45^+^/mCD45^+^ cells in peripheral blood, spleen and bone marrow. The percentage of hCD19^+^hCD45^+^/mCD45^+^ cells represent B-ALL cells infiltrating in peripheral blood, spleen and bone marrow. The infiltration degree of B-ALL cells in bone marrow, spleen and peripheral blood of mice was different. Compared with PBS, CpG 685 could significantly inhibit the infiltration of B-ALL cells in bone marrow, spleen and peripheral blood, especially the infiltration of B-ALL cells in bone marrow (left panel). Aggregated results detected from 5 B-ALL PDX mice of each group and are presented as the mean ± SD (right panel). *p < 0.05, **p < 0.01. **E** Combination therapy with CpG 685 and imatinib prolonged the survival of Sup-B15 cell egraftment in NCG mouse xenograft models. Survival curve of Sup-B15 mice of each group (7 mice per group). **F** The percentage of hCD19^+^hCD34^+^/mCD45^+^ cells represent Sup-B15 cells infiltrating in peripheral blood, spleen and bone marrow. Although Sup-B15 was resistant to CpG 685 alone in vitro, CpG 685 could inhibit the infiltration of Sup-B15 cells in mouse bone marrow, spleen, and peripheral blood in vivo. Besides, combined application of imatinib and CpG 685 could significantly inhibit the infiltration of Sup-B15 cells in spleen and peripheral blood, especially in bone marrow. (left panel) Aggregated results detected from 5 Sup-B15 mice of each group and are presented as the mean ± SD (right panel). *p < 0.05, **p < 0.01

To confirm the in vitro findings in vivo, we established a BLIN-1 engraftment in NCG mouse model. Flow cytometry results showed the engraftment and massive infiltration of hCD19^+^hCD45^+^/mCD45^+^ BLIN-1 cells in multiple organs, especially bone marrow and the spleen. The number of hCD19^+^hCD45^+^/mCD45^+^ BLIN-1 cells in the bone marrow, peripheral blood, and spleen of mice injected with CpG 685 were significantly decreased (Fig. 6A). Additionally, in vivo administration of CpG 685, not PBS, significantly inhibit the engraftment and survival of BLIN-1 cells in NCG mice (median survival: 32 days *vs.* 47 days, HR: 0.206, *p* = 0.0005. Figure 6B).

Similarly, we sorted primary Ph^−^ B-ALL cells (Additional file 1: Figure S5) with C-MYC overexpression and without BAX deletion to construct a B-ALL patient-derived tumor xenograft (PDX) model. Results showed that CpG 685 not only prolonged the survival time of PDX mice (Fig. 6C) but also reduced B-ALL burden, especially in bone marrow (Fig. 6D).

For Ph^+^ B-ALL cells, although Sup-B15 was resistant to CpG 685 alone in vitro, CpG 685 monotherapy could prolong the survival time of Sup-B15-engrafted NCG mice. Combined use of CpG 685 and imatinib further prolonged the survival time of these mice (Fig. 6E, Additional file 1: Figure S4D). Although CpG 685 monotherapy inhibited B-ALL burden in Sup-B15-engrafted NCG mice, the combination with imatinib further reduced Sup-B15 cells in mouse bone marrow, spleen, and peripheral blood (Fig. 6F).

## Discussion

Immunotherapy provides a novel strategy against B-ALL relapse and drug resistance. However, the safety and effectiveness of their application still need attention. As an immune adjuvant, CpG ODN has shown good efficacy in removing B-ALL MRD by promoting anticancer immunity and is superior to other TLR agonists [5]. However, the direct effect of CpG ODNs on B-ALL is still lacking in relevant studies. Based on this, our study systematically explored TLR9 expression in B-ALL, providing insight into the direct effects of CpG ODNs in B-ALL with different characteristics, and proposed TLR9 as a potential target for B-ALL treatment.

The direct effect of CpG ODNs on cells depends on TLR9 expression [36]. Although previous studies have found that the expression levels of TLRs in B-ALL patients are heterogeneous among different cell types [37], TLR9 expression in B-ALL has not been systematically described. Additionally, although our results were biased and needed more exploration by expanding the sample size of clinical studies, it can also provide some theoretical basis for our future research directions. Clarifying the reactivity and mechanisms of CpG ODNs for different types of B-ALL patients is also helpful for exploring the treatment strategies and sensitive population characteristics on B-ALL.

**Fig. 7:**
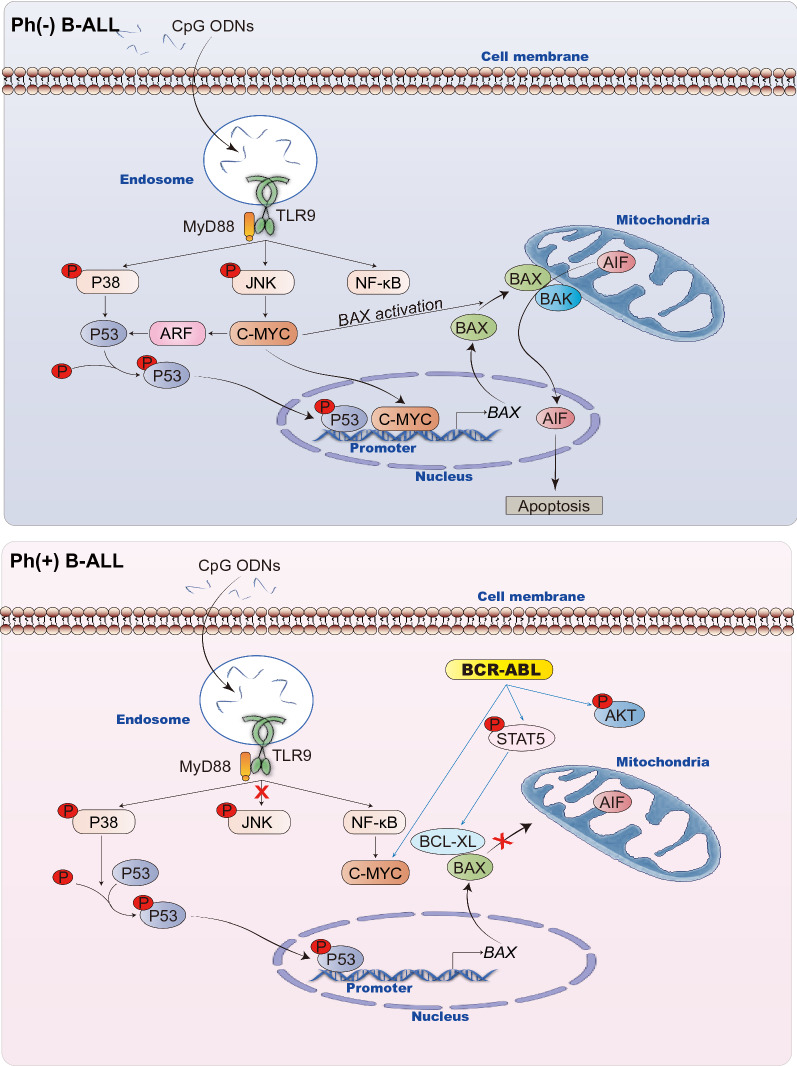
Mechanism schematic diagram of CpG 685 action in B­ALL subtypes. Mechanism of CpG 685 action in B­ALL subtypes. CpG 685 induced apoptosis in Ph^−^ B-ALL cells via TLR9 expression. It activated P38/P53/BAX and JNK/C-MYC pathways. Importantly, C-MYC overexpression on B-ALLs promoted BAX transcription as well as activation, and ARF expression. ARF expression stabilized P53, and further enhanced P53-dependent apoptosis in B-ALLs under CpG 685 stimulation. Besides, BAX activation promoted the translocation of AIF from mitochondria to the nucleus and induce caspase-independent apoptosis on Ph^−^ B-ALL. (up panel) In Ph^+^ B-ALL, due to the different downstream regulation of C-MYC, C-MYC is related to the anti-apoptotic effect. In addition, BCR-ABL inhibits BAX activation through multiple pathways, including the upregulation of BCL-XL. BCL-XL blocked BAX translocation, which further inhibited the apoptosis induced by BAX activation. (down panel) In sum, the C-MYC function and the presence or absence of BAX activation are the key to the role of CpG 685 in B-ALL

To elucidate the apoptotic signaling pathways of B-ALL mediated by CpG ODNs, we showed that TLR9 agonists induced apoptosis in B-ALL cells through P38/P53/BAX signaling, the mechanism of which depended on C-MYC-mediated BAX activation. C-MYC has crucial functions in cell growth control, differentiation, and apoptosis [38]. A previous study has shown that JNK and its downstream C-MYC-mediated pathway play important roles in the proliferation of B-cell malignancies and mainly promote cell death by activating mitochondrial apoptotic pathways [24]. C-MYC-mediated apoptosis requires both C-MYC overexpression and other pro-apoptotic insults, especially P53-dependent damage [39, 40]. P53, a universally expressed transcription factor, can be activated under stress conditions [41]. Activated P38 MAPK regulates the transcription of downstream targets by phosphorylating P53 on Ser46 and further inducing apoptosis [42, 43]. C-MYC promotes cell growth rather than apoptosis without P53 activation [44]. Definitely, the downstream target gene of overexpressed C-MYC is the key to its pro-apoptotic effect. For example, overexpressed C-MYC induces apoptosis by promoting the expression of ARF, which can stabilize P53 through MDM2 degradation [39, 45, 46]. C-MYC/MAX heterodimers can directly bind E-box elements in the *BAX* promoter, eventually promoting BAX expression [47]. C-MYC overexpression also mediates conformational changes associated with BAX activation [48]. Thus, clarifying the critical role of C-MYC in the effect of CpG 685 on B-ALL cells and its regulatory mechanism is the key to optimizing CpG 685-based precision therapy against B-ALL. In our study, by comparing the molecular mechanisms of CpG 685 apoptotic effects in four B-ALL cell lines, we clearly show that the apoptotic role of C-MYC depends on C-MYC overexpression and its downstream regulated target genes. The regulation of C-MYC on BAX is the key to CpG-induced apoptosis of B-ALL cells. Certainly, other drugs or karyotypes in B-ALL that affect BAX activation may also affect the therapeutic effect of TLR9 agonists on B-ALL. Furthermore, it was also confirmed by a B-ALL PDX model that TLR9 agonist monotherapy may be effective against Ph^−^ B-ALL with C-MYC overexpression and without BAX deletion. (Fig. 7).

Moreover, activation of bypassing pathways is one of the causes of drug resistance in tumor cells. In Ph^+^ B-ALL cells, BCR-ABL kinase blocks BAX activation through multiple pathways, leading to CpG 685 resistance. After imatinib pretreatment, inhibition of BAX activation by BCR-ABL kinase and by C-MYC was observed, and CpG 685 resistance was reversed. Thus, this study discovered the possible mechanism and solution of CpG ODN resistance.

TKI achieve significant therapeutic effects by blocking BCR‐ABL1 activity. However, because of the abnormal activation of bypassing pathways, monotherapy with either the first-generation ABL TKI imatinib or the second-generation ABL TKI dasatinib showed only a transient response to Ph^+^ B-ALL [49]. Based on this finding, exploring new therapeutic targets and clarifying their mechanism of activation may solve the problem of TKI resistance in Ph^+^ B-ALL. Interestingly, Sup-B15 was not only the primary resistant cell line of imatinib caused by BCR-ABL1-independent AKT phosphorylation [34] but also the primary resistant cell line of dasatinib due to abnormal JNK activation[50]. Our study found that CpG 685 can downregulate the levels of phosphorylated AKT and phosphorylated JNK1 in Sup-B15, which may provide combined treatment strategies against TKI resistance in Ph^+^ B-ALL.

In addition to directly promoting apoptosis by activating TLR9 downstream signaling pathways, CpG 685 increases immunogenicity on B-ALL. Similar to CpG ODN effects in B cells, TLR9 agonists upregulated CD40 and CD80 in Ph^−^ B-ALL with C-MYC overexpression. Furthermore, MHC-I upregulation could enhance recognition by CD8^+^ T cells. Although the use of CpG 685 alone in Sup-B15 did not improve its immunogenicity, CpG 685 monotherapy also inhibited B-ALL burden in Sup-B15-engrafted NCG mice (Fig. 6F). This also suggested that CpG 685 might play an anti-cancer role through immune cells such as dendritic cells and macrophages. Despite the fact that the combined use of CpG ODN and imatinib significantly upregulated CD40, MHC-I, and PD-L1 expression (Additional file 1: Figure S4C), the toxic and side effects of the combination still needed to be further explored in future studies (Additional file 1: Figure S4E). Owing to PD-L1 increases, adding PD-L1 antibodies to the combined therapy will require more studies before application in clinical practice.

In summary, CpG ODNs may represent novel therapeutic agents with multiple effects against some B-ALL subtypes, and the cell type must be considered in their application. The potential efficacy of CpG ODNs against B-ALL was confirmed in vivo and in vitro. In vivo experiments also showed a good clearance effect on B-ALL MRD. These results provided a possibility for CpG ODNs to be applied in clinic and to prolong patient survival in the future. The discovery of the roles of C-MYC and the Ph chromosome in CpG ODN treatment of B-ALL not only clarifies the mechanisms of CpG ODN activity against different subtypes but also identifies biomarkers for treatment. Owing to the different drug resistance mechanisms to CpG ODN and imatinib, combined application of CpG ODNs and other chemotherapy drugs may be a promising research direction.

## Methods

The detailed methods are provided in the Additional file data.

### Cell preparation and cell culture

Peripheral blood mononuclear cells (PBMCs) for determination of *TLR9* mRNA expression were collected from 10 healthy people and 49 patients with untreated B-ALL at The First Hospital of Jilin University between May 2013 and March 2016. To prove the effectiveness of CpG, PBMCs were collected from 14 B-ALL patients and 3 healthy controls from May to August 2019 (Additional file 1: Table S5). The patients were diagnosed (23 Ph^+^ B-ALL, 22 normal-chromosome B-ALL, and 10 with other mutations) according to the 2008 WHO classification of hematopoietic and lymphoid tissue tumors. All patients provided written, informed consent, and the study was approved by the ethics committee of The First Hospital of Jilin University (No. 2016–369).

BLIN-1 were obtained from Dr. Wei Chen (University of Minnesota, Minneapolis, MN, USA). RS4;11, NALM-6, and Sup-B15 were obtained from the American Type Culture Collection (Manassas, VA, USA). The methods of cell preparation and cell culture are shown in the Additional file data and are similar to the methods used previously reported by Wei Chen’s team (University of Minnesota, Minneapolis, MN, USA) [51].

### CpG oligodeoxynucleotides

Phosphorothioated unmethylated type-B CpG ODNs: CpG 685: 5′-TCGTCGACGTCGTTCGTTCTC-3′, was synthesized by SBI Biotech, Ltd. (Tokyo, Japan) and obtained from Dr. Wei Chen (University of Minnesota). Type-B CpG 684: 5′-TCGACGTTCGTCGTTCGTCGTTC-3′, type-B CpG 1018: 5′-TGACTGTGAACGTTCGAGATGA-3′, and type-C CpG ODNs: CpG 2395: 5′-TCGTCGTTTTCGGCGCGCGCCG-3′, were synthesized by SBI Biotech, Ltd. (Tokyo, Japan) and obtained from Dr. Jingtao Chen (Jilin University). CpG ODNs were resuspended in TE buffer (10 mmol/L Tris, 1 mmol/L EDTA, pH 8.0, T1120; Solarbio, Beijing, China) using pyrogen-free reagents, diluted in ice-cold PBS, and tested at a final concentration of 5 μg/mL or indicated doses.

### Quantitation of *TLR9* expression and miR-21

Due to the unknown expression of TLR9 in B-ALL cells, we selected patients whose B-ALL cells accounted for more than 50% of PBMCs at baseline. Detection of TLR9 expression in PBMCs in patients with B-ALL cells accounted for more than 50% of PBMCs can maximize the exclusion of TLR9 expression in other cells, such as normal B cells (Additional file 1: Table S1). Among them, TLR9 protein expression levels on B-ALL cells were detected by flow cytometry in 9 fresh B-ALL cell samples. Results confirmed that RT-qPCR results of PBMC can reflect the expression of TLR9 in B-ALL cells (Additional file 1: Figure S1B). *TLR9* expression was analyzed via qPCR and quantitated by normalizing ΔΔCt values to that of β-actin. miR-21 was quantitated by normalizing ΔΔCt values to that of U6. The action system and primer sequence are shown in the Additional file data.

To analyze TLR9 expression, cells were stained intracellularly with fluorescent antibodies against CD289 and IgG2aΚ isotype. To examine the immunostimulatory effects of CpG 685, immunoregulatory molecules were analyzed by staining with fluorescent antibodies (Additional file data). Apoptotic cells were identified with Annexin-V-PE and 7-amino-actinomycin D (7-AAD) in darkness. Cell suspensions were analyzed using a FACSCalibur flow cytometer (BD Biosciences, San Jose, CA, USA).

### siRNA transfection

80 nM negative control and C-MYC siRNA (4392420; Thermo Fisher Scientific, Inc.) were transfected into BLIN-1 cells with jetPRIME reagent (Polyplus Transfection, NY, USA) according to the manufacturer’s protocol. siRNA transfection efficiency was quantified by RT-qPCR after 48 h. The protein expression levels of C-MYC and the downregulatory molecules of C-MYC was detected by western blotting after 3-day transfection.

### Immunoblot analysis

As previously described [52], 25 mg of protein was separated on sodium dodecyl sulfate polyacrylamide (SDS-PAGE) gel and then transferred to a polyvinylidene fluoride membrane (IPVH00010; EMD Millipore, Billerica, MA, USA). Cytoplasmic and nuclei isolation for testing the nuclear translocation of apoptosis-inducing factor (AIF) were conducted as previously described [53]. Protein expression levels were detected by ECL Ultra (P10300; NCM Biotech, Newport, RI, USA) and a chemiluminescence imaging system (CLINIX, Shanghai, China). Antibodies and pathway inhibitors are shown in the Additional file data.

### Co-immunoprecipitation

Activated Bcl-2 associated X-protein (BAX) was detected via co-immunoprecipitation of BAX (6A7). Next, 50 μL protein A/G agarose (SC-2003; Santa Cruz Biotechnology, Inc., Dallas, TX, USA) was incubated with 5 μL antibody against BAX (6A7) (B8429-0.2ML; Sigma Aldrich, St. Louis, MO, USA) overnight at 4 °C. Thereafter, 250 μL total protein (1 μg/μL) was added to 55 μL mixture of each sample, followed by incubation overnight at 4 °C. Complexes were centrifuged at 6000 ×*g* for 10 min at 4 °C and washed twice with sample buffer. Finally, the beads were resuspended in SDS sample buffer and boiled for 10 min before western blotting.

### Immunofluorescence and laser scanning confocal microscopy

To clarify the expression of BAX (6A7) and co-localization with mitochondria, we treated cells with 150 nM Mito Tracker Red CMXRos (9082; Cell Signaling Technology, Danvers, MA, USA) for 30 min at 37 °C. Cells were washed twice with phosphate-buffered saline (PBS), followed by fixation in 4% paraformaldehyde at 4 °C for 10 min and in pre-cooled methanol at − 20 °C for 20 min. Then, the fixed cells were centrifuged at 1800 ×*g* for 15 min onto a glass slide. The other steps are consistent with a previously reported protocol [54].

### Chromatin immunoprecipitation (ChIP)

B-ALL cells were treated with CpG 685 or PBS for 12 h, harvested, and processed as we reported previously [55]. Finally, the E-box regions of the *BAX* promoter were analyzed by real-time qPCR. The primers used were in accordance with a previously reported study [47] and are listed in the Additional file: data.

### NCG mouse xenograft models

6-to-8-week-old male/female NCG mice were purchased from Nanjing Biomedical Research Institute of Nanjing University and were housed in a specific pathogen-free micro-isolator environment at the First Hospital Animal Center of Jilin University. The study was approved by the ethics committee of The First Hospital of Jilin University (No. 2020–0480). The methods were similar to those reported previously [51, 56] and are detailed as follows.

BLIN-1 xenotransplantation NCG mouse model: To determine the engraftment and tumorgenicity of BLIN-1 cells in vivo, mice were i.v. injected with 5 × 10^6^ BLIN-1 cells. After 15 days, cohorts of mice were also i.p. injected with 2 mg/kg CpG 685 or PBS control for the next 5 days. On day 30, flow cytometry was used to detect the percentage of hCD19^+^hCD45^+^/mCD45^+^ cells in peripheral blood, spleen and bone marrow. Besides, to observe CpG 685-mediated anti-tumor efficacy in vivo, cohorts of mice (n = 7 for each cohort) were monitored for the survival time.

Sup-B15 xenotransplantation NCG mouse model: To determine the engraftment and tumorgenicity of Sup-B15 cells in vivo, mice were i.v. injected with 2 × 10^7^ Sup-B15 cells. After 15 days, cohorts of mice were also i.p. injected with 2 mg/kg CpG 685 or/and 50 mg/kg imatinib for the next 5 days. On day 30, flow cytometry was used to detect the percentage of hCD19^+^hCD34^+^/mCD45^+^ cells in peripheral blood, spleen and bone marrow. Besides, to observe CpG 685-mediated anti-tumor efficacy in vivo, cohorts of mice (n = 7 for each cohort) were monitored for the survival time.

B-ALL PDX mouse model: Cohorts of mice were i.v. injected with 1 × 10^6^ primary B-ALL cells (patient inflammation in Additional file 1: Figure S5). At days 10, blood is taken through the tail vein to check the tumor burden. According to the proportion of hCD19^+^hCD45^+^/mCD45^+^ cells in peripheral blood, PDX model mice were divided into two groups. Each cohort of mice were also i.p. injected with 2 mg/kg CpG 685 or PBS control for the next 5 days. On day 22, flow cytometry was used to detect the percentage of hCD19^+^hCD45^+^/mCD45^+^ cells in peripheral blood, spleen and bone marrow. Besides, to observe CpG 685-mediated anti-tumor efficacy in vivo, cohorts of mice (n = 7 for each cohort) were monitored for the survival time.

### Data analysis

Data were analyzed using SPSS 24.0 (SPSS, Inc., Chicago, IL, USA). More details are provided in the Additional file data. Statistical significance was determined using unpaired two-tailed Student’s *t*-test. Numeric data are presented as mean ± standard deviation (SD) or mean ± structural equation modeling (SEM). *p < 0.05, **p < 0.01.

## Supplementary Information

**Additional file 1. **Additional tables S1–S5 and additional figures S1–S5.

## Data Availability

Clinicopathological features of B-ALL patients and healthy controls can be found in the Additional file: data available in the online version of this article. For original data, please contact lingbai@jlu.edu.cn.
