# Supplementary material for: BAX as the mediator of C-MYC sensitizes acute lymphoblastic leukemia to TLR9 agonists
Source: J Transl Med. 2023 Feb 10;21:108. doi: 10.1186/s12967-023-03969-z (PMC9921080; doi:10.1186/s12967-023-03969-z)
Supplement: Supplementary file 1 — Additional file 1. Additional tables S1–S5 and additional figures S1–S5. [file 12967_2023_3969_MOESM1_ESM.pdf]

Additional file data

Additional file data on materials and methods

Cell preparation and cell cultures

PBMCs were isolated using Ficoll-Paque (17-1440-03; GE Healthcare, Uppsala, Sweden). At clinical diagnosis, molecular diagnosis were confirmed via RT-PCR and fluorescence in situ hybridization. PBMC cultured in X-VIVO 15 serum-free medium (04-744Q; Lonza Walkersville, MD, USA) with 10% FBS (10099-141; Thermo Fisher Scientific, Waltham, MA, USA). BLIN-1 cells maintained in 10% FBS RPMI 1640 media (10099-141; Thermo Fisher Scientific). And Sup-B15 cells cultured in IMDM media with 20% FBS.

To measure the proliferation of B-ALL cells stimulated by CpG 685, BLIN-1 and Sup-B15 cells were cultured in media with different doses (1, 2, 5, and 10 μg/mL) of CpG 685 at 4 × 104 cells/well in 96-well plates. Each group was provided with 5 duplicate wells. After 3 days, cells were analyzed by a WST-1 cell viability assay (11644807001; Roche, Basel, Switzerland). To determine the effects of CpG 685 on apoptosis, the cell cycle and the immune stimulatory effects in B-ALL cells, the two cell lines were cultured in media with different doses of CpG 685 at 1 × 106 cells/well in 6-well plates. Cells receiving no CpG 685 treatment were used as a control. Each group was provided 3 duplicate wells. At the end of each treatment time point, cells were harvested and assessed. All B-ALL cells were cultured in complete medium in a 5% CO_2-humidified incubator at 37 °C.

Real-time PCR action system

Total RNA was extracted using the Eastep Super Total RNA Extraction Kit (LS1040; Promega, Madison, WI, USA) according to the manufacturer's protocol. Complementary DNA (cDNA) was synthesized using the Primescript RT reagent kit with a gDNA Eraser (RR047A; Takara, Kyoto, Japan) from total RNA. PCR primers for *TLR9* introns were used to exclude RNA samples mixed with cDNA introns. To quantify miRNA using qPCR, a Mir-X miRNA first-stand synthesis kit (638315; Takara Bio USA, Inc., Mountain View CA, USA) was used to synthesize cDNA for miR-21 qPCR.

ddH2O 3.5 µL, forward primer 0.5 µL, reverse primer 0.5 µL, cDNA 0.5 µL, SYBR Green Master 5 µL (04913914001; Roche, Basel, Switzerland). E-box 1, 2, 3, and 4 of Bax as well as TLR9 PCR parameters were as follows: 95 ℃ 10 min, 65 ℃ 5 s, 95 ℃ 50 s; 40 cycles: 95 ℃ 10 s, 60 ℃ 20 s. miR-21 PCR parameters were as follows: 95 ℃ 10 s, 95 ℃ 60 s, 55 ℃ 30 s, 95℃ 30 s; 40 cycles: 95 ℃ 5 s, 60 ℃ 20 s.

Primers

All primers were manufactured by Comate Bioscience Corporation (Changchun, China). The primer sequences (5′-3′) were as follows: *β-actin* forward primer, CAGGTCATCACCATTGGCAATGAGC; *β-actin* reverse primer, CGGATGTCCACGTCACACACTTCATGA; *TLR9* forward primer, TGCAGCCGGAGATGTTTG; *TLR9* reverse primer, CGCGGTAGCTCCGTGAAT; *TLR9* intron forward primer, ATTAAGGCAAAGGCTCTCGGG; *TLR9* intron reverse primer, AGCTCTACCTCCACCCACTC. *C-myc* forward primer, CTTCTCTCCGTCCTCGGATTCT; *C-myc* reverse primer, GAAGGTGATCCAGACTCTGACCTT. E-box 1 forward primer, GCTCATTGGACAGTGTGCA; E-box 1 reverse primer, CACGGGACCAAACCTC; E-box 2 forward primer, GAGGCAGGTGCGGTGTGCACTC; E-box 2 reverse primer, CCGGCGGCGCTGC; E-box 3 and 4 forward primer,

TTTTGCGGGGCGGCGTGCACAAG; E-box 3 and 4 reverse primer, CCGGGCGCGCTGC. *miR-21*-specific primer, GCCCGCTAGCTTATCAGACTGATG.

Fluorescent antibody

CD289 (17-9099-82; Thermo Fisher Scientific, Waltham, MA, USA), IgG2aK isotype (17-4321-81; Thermo Fisher Scientific), Annexin-V-PE (12-8102-69; eBioscience, Thermo Fisher Scientific, Waltham, MA, USA), 7-amino-actinomycin D (7-AAD) (00-6993-42; eBioscience), CD40 (17-0409-42; Thermo Fisher Scientific), CD80 (340294; BD Biosciences, Franklin Lakes, NJ. USA), CD86 (555657; BD Biosciences), MHC-Ⅰ (17-9958-42; Thermo Fisher Scientific), HLA-DR (560744; BD Biosciences), Fas (561655; BD Biosciences), FasL (306407; BioLegend, San Diego, CA, USA), DR4 (307206; BioLegend), DR5 (FAB6311P; R&D Systems, Foster City, CA, USA), MICA (FAB1300P; R&D Systems), MICB (FAB1599A; R&D Systems), PD-L1 (329708; BioLegend, San Diego, CA, USA), hCD45 (368508; BioLegend), hCD19 (302207; BioLegend), hCD34 (343504; BioLegend), Aqua (423101; BioLegend), mCD45 (103112; BioLegend), and isotype control antibodies.

Primary antibodies for western blot

β-actin (1:1000, AA128; Beyotime Biotechnology), P65 (1:1000, CS- 8242; Cell Signaling Technology; Danvers, MA, USA), P-P65 (1:1000, CS-3033; Cell Signaling Technology), P38 (1:1000, 8690; Cell Signaling Technology), P-P38 (1:500, CS-4937; Cell Signaling Technology), JNK (1:1000, SC-9252; Santa Cruz Biotechnology, Dallas, TX, USA), P-JNK (1:500, CS-9255; Cell Signaling Technology), P-P53 (ser46; 1:500, ab76242; Abcam, Cambridge, UK), P53 (1:500, SC-6243; Santa Cruz Biotechnology), C-MYC (1:1000, ab32072; Abcam), ARF (1:500, SC-53639; Santa Cruz Biotechnology, Dallas, TX, USA), BAX (1:1000, 50599-2-IG; Thermo Fisher Scientific), BAK (1:1000, ab32371;

Abcam), cleaved PARP (1:1000, CS-5625; Cell Signaling Technology), AKT (1:1000, CS-4691; Cell Signaling Technology), P-AKT (ser473; 1:1000, CS-4060; Cell Signaling Technology), PTEN (1:1000, ab32199; Abcam), BCL-XL (1:1000, ab32370; Abcam), P-STAT5 (1:1000, CS-4322; Cell Signaling Technology), AIF (1:1000, ab5318; Abcam), GAPDH (1:3000, ab8245; Abcam), and Histone H3 (1:1000, ab1791; Abcam).

Signaling pathway activation confirmation

To block the p38 mitogen-activated protein kinase (p38 MAPK) pathway, JNK pathway, C-MYC pathway, BAX activation, Caspase pathway, and BCR-ABL kinase, Cells were pretreated with 40 μM SB203580 (1EA S8307-1MG; Sigma Aldrich, St. Louis, MO, USA) for 30 min, 40 μM SP600125 (S1460; Selleck Chemicals, Houston, TX, USA) for 60 min, different doses of 10058-F4 (F3680-5MG; Sigma Aldrich) for 60 min, 0.5 μM BAI1 for 60 min, 80 μM Quinoline-Val-Asp-Difluorophenoxymethylketone (Q-VD-OPh) for 60min, and different doses of imatinib (CDS022173-25MG; Sigma Aldrich) for 3 h, respectively. The cells were then cultured in media with or without CpG 685. At the end of each time point, cells were harvested and assessed by western blotting.

Data analysis

Clinical data were analyzed using SPSS 24.0 (SPSS, Inc., Chicago, IL, USA). For univariate analysis of continuous variables, the Mann-Whitney U test or Kruskal-Wallis H test was used. For multivariate analysis of continuous variables, linear regression analysis was applied. For categorical variables, Pearson's χ^2 test or Fisher's exact test was used. For survival analysis, the log-rank test was used for comparison in univariate analysis. Cox multivariate model was used in multivariate analysis to calculate hazard ratio (HR) and 95% confidence interval (CI).

The Kaplan-Meier method was used to evaluate the progression-free survival (PFS) and overall survival (OS) of the two groups. For comparison of the viable B-ALL cells in clinical samples with or without CpG 685, two-tailed paired *t*-test was used.

To determine the difference between protein expression levels in the CpG 685-treated and untreated groups, relative expression levels were calculated using ImageJ (NIH, Bethesda, MD, USA) in three independent experiments and analyzed using GraphPad Prism 7.0a for Mac OS X (GraphPad Inc., La Jolla, CA, USA). Statistical significance was determined using the unpaired two-tailed Student's *t*-test. Numeric data are presented as mean ± standard deviation (SD).

Additional file 1: Table S1. Clinicopathological features of B-ALL patients and healthy people used in the analysis of TLR9 expression levels.

Accession number	Sex	Age (years)	WBC count ($\times 10^9$/L)	B-ALL (%)	Immunophenotype	Cytogenetics	Complete Remission	PFS (months)	PFS censored sample	OS (months)	OS censored sample	Treatment or not	TLR9 expression
**T-20**	M	60	3.48	95.5	Common precursor-B	47,XX,6q-,+10	-	-	Yes	-	Yes	Yes	0.22714879
**T-24**	F	24	3.5	61	Pro-B	49,XY,+X,+2,+8,del(11q),ddl(22q)	＜4 weeks	49	No	49	Yes	Yes	0.06763356
**T-26**	F	33	411.72	83	Pre-B	t(4;11)(q21,q23)	＜4 weeks	3	No	6	No	Yes	0.31425179
**T-28**	F	20	103.99	80	Common precursor-B	t(9;22)(q34,q11)	＜4 weeks	9.5	No	9.5	No	Yes	0.1429798

**T-31**	M	40	260.14	52	Common precursor-B	t(9;22)(q34,q11)	＜4 weeks	5	No	23	No	Yes	0.11430508
**T-32**	F	58	148.21	57	Common precursor-B	t(9;22)(q34,q11)	＜4 weeks	6	No	15	No	Yes	0.77088021
**T-49**	F	15	55.35	56	Common precursor-B	Normal chromosome	-	0.8	No	0.8	No	No	0.09271093
**T-57**	M	3	27.47	65	Common precursor-B	Normal chromosome	＜4 weeks	24	Yes	24	Yes	Yes	0.10543731
**T-61**	F	3	4.2	90.5	Common precursor-B	TEL-AML1	＜4 weeks	26	Yes	26	Yes	Yes	0.52756553
**T-63**	F	51	38.83	84	Common precursor-B	t(9;22)(q34,q11)	-	1	No	1	No	No	0.06733112
**T-64**	F	12	636.1	91	Common precursor-B	Normal chromosome	≥4 weeks	1.5	No	1.5	No	Yes	0.09476016
**T-65**	F	11	229.23	78	Common precursor-B	t(9;22)(q34,q11)	≥4 weeks	15	Yes	15	Yes	Yes	2.97934023
**T-66**	F	5	2.65	24	Common precursor-B	Normal chromosome	＜4 weeks	24	Yes	24	Yes	Yes	0.05886129
**T-68**	F	26	90.57	86	Common precursor-B	t(9;22)(q34,q11)	＜4 weeks	17	No	22	Yes	Yes	3.55535319
**T-73**	M	2	4.29	96	Common precursor-B	Normal chromosome	≥4 weeks	14	No	14	No	Yes	0.25602693
**T-74**	M	39	63.02	69	Common precursor-B	t(9;22)(q34,q11)	-	4	No	10	No	Yes	0.1234736

**T-79**	F	43	21.2	70	Common precursor-B	Normal chromosome	＜4 weeks	6	No	6	No	Yes	0.07458752
**T-80**	M	19	336.68	68	Common precursor-B	t(9;22)(q34,q11)	-	12	No	45	Yes	Yes	0.64626685
**T-81**	M	16	11.72	66	Common precursor-B	Normal chromosome	≥4 weeks	56	Yes	56	Yes	Yes	0.29335182
**T-82**	F	9	5.92	94	Common precursor-B	TEL-AML1	＜4 weeks	20	Yes	20	Yes	Yes	0.07535626
**T-83**	M	75	87.86	94	Common precursor-B	t(9;22)(q34,q11)	-	3	No	3	No	No	0.10782219
**T-84**	F	6	195.79	88	Pro-B	dupMLL	＜4 weeks	52	Yes	52	Yes	Yes	0.24762004
**T-85**	F	40	483.6	81	Common precursor-B	t(9;22)(q34,q11)	＜4 weeks	29	No	31	No	Yes	0.12248049
**T-87**	M	5	18.68	83	Common precursor-B	Normal chromosome	＜4 weeks	24	Yes	24	Yes	Yes	0.648413
**T-89**	M	20	37.68	54	Common precursor-B	Normal chromosome	＜4 weeks	57	Yes	57	Yes	Yes	0.1401466
**T-90**	M	7	190.9	83.36	Common precursor-B	t(9;22)(q34,q11)	＜4 weeks	25	Yes	25	Yes	Yes	0.09956498
**T-3**	F	33	32.23	61	Common precursor-B	Normal chromosome	＜4 weeks	9	No	-	Yes	Yes	0.00445737
**T-4**	M	40	260.14	52	Common precursor-B	t(9;22)(q34,q11)	＜4 weeks	5	No	23	No	Yes	0.00381673
**T-25**	M	45	13.98	41	Pro-B	Normal chromosome	-	-	Yes	1	No	No	0.00370737

**T-29**	M	40	52.51	63	Common precursor-B	t(9;22)(q34,q11)	＜4 weeks	7	No	13	No	Yes	0.01551791
**T-39**	F	51	92.63	72	Common precursor-B	Normal chromosome	-	1	No	1	No	Yes	0.01403543
**T-40**	M	35	6.68	65	Common precursor-B	Normal chromosome	＜4 weeks	12	No	21	No	Yes	0.00867205
**T-42**	M	39	20.2	56	Common precursor-B	Normal chromosome	＜4 weeks	5	No	12	No	Yes	0.01160749
**T-43**	M	73	1.22	63.5	Common precursor-B	Normal chromosome	-	-	Yes	1	No	No	0.03355761
**T-47**	M	18	3.44	89	Common precursor-B	Normal chromosome	＜4 weeks	74	Yes	74	Yes	Yes	0.01915617
**T-48**	F	20	1.74	90	Common precursor-B	t(9;22)(q34,q11)	-	1	No	1	No	No	0.01996066
**T-51**	M	22	31.83	70	Common precursor-B	del(3p),-6,-10,-14,+marX 3	＜4 weeks	17	No	18	No	Yes	0.03756904
**T-52**	M	33	12	41	Common precursor-B	t(9;22)(q34,q11)	＜4 weeks	56	Yes	56	Yes	Yes	0.03280437
**T-53**	M	52		-	Common precursor-B	t(9;22)(q34,q11)	-	9	No	9	No	Yes	0.02853384
**T-54**	M	45	1.79	84.5	Common precursor-B	Normal chromosome	＜4 weeks	14	No	14	No	Yes	0.01431681
**T-58**	F	45	13.37	42	Pro-B	Normal chromosome	＜4 weeks	7	No	11	No	Yes	0.02191596

**T-59**	F	63	213.87	96	Common precursor-B	t(9;22)(q34,q11)	-	3	No	12	No	Yes	0.02822516
**T-60**	M	52	45.63	86	Pro-B	t(4;11)(q21,q23)	＜4 weeks	9	No	12	No	Yes	0.02651951
**T-67**	F	42	217.42	96	Common precursor-B	t(9;22)(q34,q11)	-	-	Yes	-	Yes	No	0.02127258
**T-69**	F	26	10.36	79	Pro-B	Normal chromosome	＜4 weeks	14	No	18	No	Yes	0.03189369
**T-71**	F	14	47.82	29	Pro-B	Normal chromosome	-	-	Yes	-	Yes	Yes	0
**T-77**	F	2	72.17	90	Common precursor-B	Normal chromosome	＜4 weeks	16	Yes	16	Yes	Yes	0
**T-78**	F	47	46.15	62	Common precursor-B	t(9;22)(q34,q11)	-	0.5	No	0.5	No	No	0.05851842
**T-88**	M	20	89.98	47	Common precursor-B	Normal chromosome	≥4 weeks	21	No	25	No	Yes	0.02734448
**N-1**	F	47	7.19	-	-	-	-	-	-	-	-	-	0.01122729
**N-2**	M	32	4.78	-	-	-	-	-	-	-	-	-	0.00036284
**N-3**	F	12	8.2	-	-	-	-	-	-	-	-	-	0
**N-4**	M	48	4.57	-	-	-	-	-	-	-	-	-	0.00192442
**N-5**	F	26	5.68	-	-	-	-	-	-	-	-	-	0.00034159
**N-6**	F	8	9.23	-	-	-	-	-	-	-	-	-	0
**N-7**	M	22	6.45	-	-	-	-	-	-	-	-	-	0.01021425
**N-8**	M	52	5.62	-	-	-	-	-	-	-	-	-	0.00348217
**N-9**	F	65	5.34	-	-	-	-	-	-	-	-	-	0
**N-10**	M	19	6.79	-	-	-	-	-	-	-	-	-	0

Additional file 1: Table S2. Univariate analysis of clinical characteristics in relation to TLR9 expression.

While determining the relationship between TLR9 expression and clinical characteristics, we found that cytogenetics was correlated with TLR9 expression through single factor analysis ($p = 0.045$), while multivariate linear regression analysis did not find any difference ($p > 0.05$).

Clinical characteristics	n (%)	TLR9 expression	*p*-value
Age			0.051
＜18 years	14 (28.60)	0.103±0.281	
≥18years	35 (71.40)	0.033±0.103	
Sex			0.66
Male	25 (51.00)	0.068±0.174	
Female	24 (49.00)	0.036±0.120	
WBC count (×10^9/L)			0.503
WBC＜30	20 (41.70)	0.046±0.177	
WBC≥30	28 (58.30)	0.094±0.120	
Immunophenotype			0.135
Pro-B	7 (14.30)	0.027±0.064	
Common precursor-B	41 (83.70)	0.075±0.121	
Pre-B	1 (2.00)	0.314	
Cytogenetics			0.045*
Normal chromosome	22 (44.90)	0.030±0.087	
t(9;22)(q34,q11)	19 (38.80)	0.100±0.115	
With other genetic mutations	8 (16.30)	0.151±0.253	
Complete Remission			0.149

＜4 weeks	29 (85.30)	0.068±0.124	
⩾4 weeks	5 (14.70)	0.256±1.575	

Additional file 1: Table S3. Baseline comparison of TLR9 high and low expression groups in B-ALL patients under standard regular treatment.

Through baseline analysis of TLR9 high and low expression groups of patients under standard regular treatment following NCCN Guidelines, a difference in age distribution between the two groups was found (p = 0.018).

(Due to financial constraints, the Ph^+ B-ALL patients were treated only intermittently with TKI.)

Clinical characteristics	TLR9 high group (N=23)	TLR9 low group (N=18)	p-value
Age			0.018*
＜18 years	11 (47.8%)	2 (11.1%)	
⩾18years	12 (52.2%)	16 (88.9%)	
Sex			0.262
Male	13 (56.5%)	7 (38.9%)	
Female	10 (43.5%)	11 (61.1%)	
WBC count ($\times 10^9$/L)			0.884
WBC＜30	10 (43.5%)	10 (58.8%)	
WBC⩾30	13 (56.5%)	7 (41.2%)	
Immunophenotype			0.342
Pro-B	2 (8.7%)	4 (22.2%)	
Common precursor-B	20 (87.0%)	14 (77.8%)	
Pre-B	1 (4.3%)	0 (0%)	
Cytogenetics			0.217
Normal chromosome	8 (34.8%)	11 (61.1%)	
t(9;22)(q34,q11)	9 (39.1%)	5 (27.8%)	
With other genetic mutations	6 (26.1%)	2 (11.1%)	
Complete Remission			0.379
＜4 weeks	16 (69.6%)	13 (72.2%)	
⩾4 weeks	4 (17.4%)	1 (7.1%)	

Additional file 1: Table S4. Univariate analysis of clinical characteristics of PFS and OS in B-ALL patients under standard regular treatment.

Univariate analysis suggests that age, white blood cell count, immunophenotype, and TLR9 expression levels may be the independent prognostic factors of PFS; age, immunophenotype, and TLR9 expression levels may be the independent prognostic factors of OS ($p < 0.1$).

(Due to financial constraints, the Ph^+ B-ALL patients were treated only intermittently with TKI.)

Clinical characteristics	n (%)	Univariate analysis of PFS			Univariate analysis of OS		
		HR	95% Cl	*p*-value	HR	95% Cl	*p*-value
Age		8.258	1.939-35.180	0.004*	5.799	1.352-24.873	0.018*
＜18 years	13 (31.7)						
⩾18years	28 (68.3)						
Sex		0.806	0.373-1.741	0.583	0.807	0.347-1.874	0.618
Male	20 (48.8)						
Female	21 (51.2)						
WBC count (×10^9/L)		2.341	1.005-5.454	0.049*	1.835	0.740-4.551	0.19
WBC＜30	17 (42.5)						
WBC⩾30	23 (57.5)						
Immunophenotype				0.095			0.1
Pro-B	6 (14.6)						
Common precursor-B	34 (82.9)	0.982	0.334-2.888		0.993	0.291-3.385	0.991
Pre-B	1 (2.4)	12.059	1.044-139.268		11.837	0.975-143.723	0.052

Cytogenetics							
Normal chromosome	19 (46.3)			0.365			0.751
t(9;22)(q34,q11)	14 (34.1)	1.617	0.697-3.747		1.043	0.424-2.569	
With other genetic mutations	8 (19.5)	0.786	0.249-2.475		0.639	0.176-2.328	
Complete Remission		0.935	0.275-3.182	0.914	1.247	0.360-4.321	0.728
＜4 weeks	29 (85.3)						
⩾4 weeks	5 (14.7)						
TLR9 expression		1.952	0.895-4.257	0.093	2.543	1.076-6.009	0.033*
TLR9 expression⩾0.067	23 (56.1)						
TLR9 expression<0.067	18 (43.9)						

Additional file 1: Table S5. Clinicopathological features of B-ALL patients and healthy people treated with CpG 685 or PBS.

Accession number	Sex	Age (years)	Immunophenotype	Cytogenetics	B-ALL cells percentage	TLR9 expression	C-MYC expression	BAX expression	B-ALL cell counts after 3d treatment, percentage	
									PBS	CpG 685
T-110	M	2.5	Pre-B	Normal chromosome	76.54%	8.93E-04	7.47E-12	8.46E-03	20.6	43.2
T-114	M	3	Common precursor-B	Normal chromosome	85.00%	2.58E-05	9.14E-12	9.32E-03	15.1	19
T-104	F	7	Common precursor-B	HOX11	80.80%	4.87E-05	2.22E-11	7.18E-02	14.3	26.8
T-119	M	2.5	Pre-B	Normal chromosome	37.22%	1.04E-05	2.66E-11	3.41E-03	54.4	69
T-112	M	4	Common precursor-B	Normal chromosome	80.62%	1.11E-05	3.85E-11	5.53E-02	85.4	93.5
T-103	M	39	Common precursor-B	t(9;22)(q34,q11)	85.27%	2.18E-04	5.37E-11	4.62E-02	14.6	49.8
T-113	M	6	Common precursor-B	TEL-AML1	89.76%	2.78E-04	5.76E-11	4.64E-02	13.1	9.54
T-102	F	6	Pre-B	Normal chromosome	51.52%	1.37E-06	5.83E-11	3.74E-03	39.3	73.7
T-118	M	3	Common precursor-B	Normal chromosome	94.65%	1.17E-04	1.24E-10	7.21E-02	80.2	81.4

T-115	M	9	Common precursor-B	Normal chromosome	82.68%	3.76E-04	1.34E-10	1.03E-02	4.43	16.6
T-111	F	6	Common precursor-B	TEL-AML1	91.93%	2.81E+00	6.22E-10	3.40E-04	59.6	54.6
N-013	M	12	-	-	-	3.92E-02	7.08E-10	1.15E-02	74.8	79
T-109	F	37	Common precursor-B	Normal chromosome	80.63%	4.02E-04	8.02E-10	1.31E-02	53.4	58.1
N-014	F	40	-	-	-	1.35E-03	8.15E-10	4.10E-04	26.1	45.6
N-020	M	55	-	-	-	1.37E-04	9.22E-10	2.18E-03	82.3	90.3
T-106	F	4	Common precursor-B	Normal chromosome	66.80%	7.33E-02	1.24E-09	5.62E-03	84.6	75.2
T-108	M	30	Pre-B	Normal chromosome	83.34%	4.00E-04	1.48E-09	9.54E-02	58.9	48.8

Additional file 1: Figure S1

(A) The online analysis website “The Human Protein Atlas” (https://www.proteinatlas.org/) analyzed the *TLR9* mRNA expression of 29 immune cells in the Monaco dataset, and found that *TLR9* is not only expressed in pDCs but also B cells. The expression of TLR9 in B-cell subtypes also shows distinct.

(B) TLR9 expression trend of RT-PCR is consistent with Flow. RT-PCR results were confirmed by Flow in the corresponding patients. The samples of both fresh and frozen B-ALL cells were obtained from 9 untreated patients. Flow cytometry were used to test TLR9 protein expression level. Intracellular staining results of TLR9 protein expression (red-shaded histogram) indicated with MFI number and overlaid with isotype control (grey-shaded histogram). RT-qPCR were used to modify the mRNA expression level of TLR9. β-actin was used as the standard for mRNA expression.

(C) RT-qPCR results of TLR9 mRNA expression in these 6 cell lines. TLR9 expression in BLIN-1, RS4;11, NALM-6, and Sup-B15 was determined and comparatively analyzed using Jurkat cells as the positive control and Hela cells as the low-expression control. β-actin was used as the standard for mRNA expression.

(D) CpG ODNs can induce BLIN-1 and RS4;11 apoptosis. The apoptotic effect of CpG 685 on B-ALL is stronger than other B-type CpG ODNs and C-type CpG ODNs.

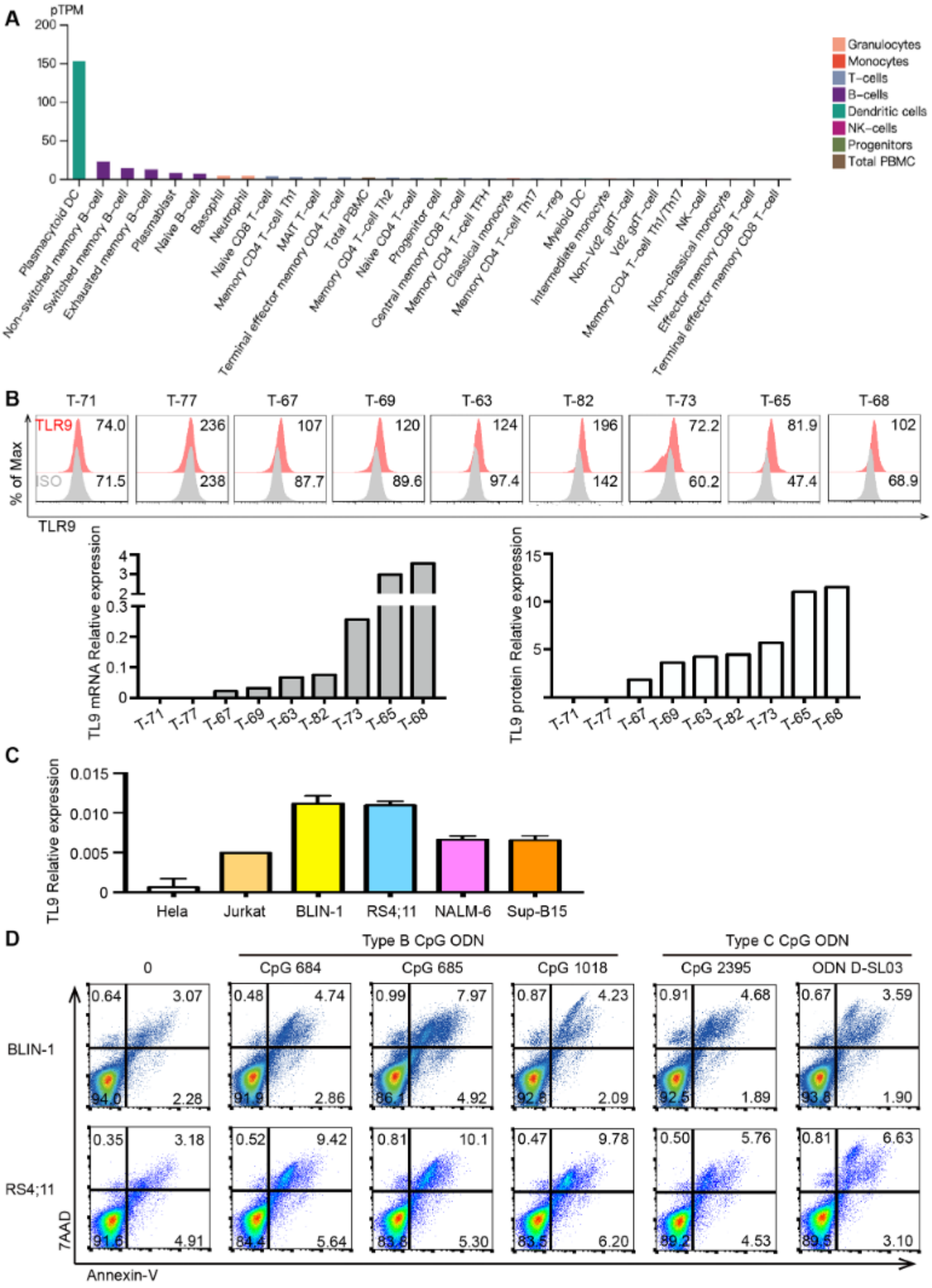
A
pTPM
200
150
100
50
0
Plasmacytoid DC
Non-switched memory B-cell
Switched memory B-cell
Exhausted memory B-cell
Plasmablast
Naive B-cell
Basophil
Neutrophil
Naive CD8 T-cell
Memory CD4 T-cell Th1
MAIT T-cell
Terminal effector memory CD4 T-cell
Total PBMC
Memory CD4 T-cell Th2
Naive CD4 T-cell
Progenitor cell
Central memory CD8 T-cell
Memory CD4 T-cell TFH
Classical monocyte
Memory CD4 T-cell Th17
T-reg
Myeloid DC
Intermediate monocyte
Non-Vd2 gdT-cell
Vd2 gdT-cell
Memory CD4 T-cell Th1/Th17
NK-cell
Non-classical monocyte
Effector memory CD8 T-cell
Terminal effector memory CD8 T-cell
Granulocytes
Monocytes
T-cells
B-cells
Dendritic cells
NK-cells
Progenitors
Total PBMC
B
T-71
T-77
T-67
T-69
T-63
T-82
T-73
T-65
T-68
TLR9
ISO
74.0
71.5
236
238
107
87.7
120
89.6
124
97.4
196
142
72.2
60.2
81.9
47.4
102
68.9
% of Max
TLR9
TL9 mRNA Relative expression
TL9 protein Relative expression
C
TL9 Relative expression
0.015
0.010
0.005
0
Hela
Jurkat
BLIN-1
RS4;11
NALM-6
Sup-B15
D
0
Type B CpG ODN
CpG 684
CpG 685
CpG 1018
Type C CpG ODN
CpG 2395
ODN D-SL03
BLIN-1
RS4;11
7AAD
Annexin-V
0.64 3.07 94.0 2.28
0.48 4.74 91.9 2.86
0.99 7.97 86.1 4.92
0.87 4.23 92.8 2.09
0.91 4.68 92.5 1.89
0.67 3.59 93.8 1.90
0.35 3.18 91.6 4.91
0.52 9.42 84.4 5.64
0.81 10.1 83.8 5.30
0.47 9.78 83.5 6.20
0.50 5.76 89.2 4.53
0.81 6.63 89.5 3.10

Additional file 1: Figure S2

(A) Flow cytometry results showing kinetic changes in BLIN-1 and Sup-B15 cell number after 3-day culture in media with different doses of CpG 685 (left panel). With the prolongation of B-ALL stimulation time, CpG 685 could further promote BLIN-1 cell apoptosis. At 7 days, compared with PBS control, 5 μg/mL CpG 685 could promote BLIN-1 cell apoptosis nearly 30 times (right panel).

(B) Schematic diagram of *ba*x promoter-CAT reporter construct (left panel). Position +1 refers to the *bax* translational start site.

(C) Other surface molecules expression on BLIN-1 with (red-shaded histogram) or without (dark gray-histogram) 5 μg/mL CpG 685 after 24 h culture.

(D) The WST-1 cell viability assay showed dynamic changes to BLIN-1 cell number with different doses of 10058-F4 pretreatment cultured in media with or without 5 μg/ml CpG 685 for 24 h. 10058-F4 at the concentration of 25 μM or above can blocked the BLIN-1 apoptosis induced by CpG 685. Columns represent means of 5 independent experiments; bars represent SD. **p*<0.05, ***p*<0.01.

(E) The percentage of Annexin-V^+ cells in BLIN-1 cells (fold) which were incubated alone or in the presence of CpG 685 with or without inhibitor pretreatment for 3-day was analyzed by flow cytometry. Columns represent means of 3 independent experiments; bars represent SD. **p*<0.05, ***p*<0.01. The red line represents 1-fold.

(F) BLIN-1 cells were transfected by negative control or C-MYC siRNA after 48 h. C-MYC expression, P53 expression, activation of phosphorylated p53 at ser46, BAX expression, and PARP cleavage were tested with or without CpG 685 after 24 h by western blot. Interfering with the expression of C-MYC can block C-MYC activated by CpG 685 and its downstream signaling pathways that induce apoptosis.

(G) WST-1 cell viability assay results showing kinetic changes in B-ALL cell number after 3-day culture in media with different doses of CpG 685. Although CpG 685 could inhibit proliferation in RS4;11 cells, it had no significant effect on NALM-6 and Sup-B15 cells in vitro. Columns represent means of 5 independent experiments; bars represent SD. **p*<0.05, ***p*<0.01.

(H) CpG 685 activates downstream molecules of the TLR9 signaling pathway in RS4;11 cells. Phosphorylated P65, phosphorylated P38, phosphorylated JNK1, C-MYC, ARF, P53, phosphorylated p53 at ser46, BAX, and PARP cleavage in RS4;11

cells cultured with or without 5 μg/mL CpG 685 were examined by western blotting at the indicated time points.

(I) CpG 685 activates downstream molecules of the TLR9 signaling pathway in NALM-6 cells. NALM-6 is a B-ALL cell line with BAX deficiency. Phosphorylated P65, phosphorylated P38, phosphorylated JNK1, C-MYC, ARF, P53, phosphorylated p53 at ser46, BAX, and PARP cleavage in BLIN-1 cells cultured with or without 5 μg/mL CpG 685 were examined by western blotting at the indicated time points.

(J) Nuclear translocation of AIF is a key event of caspase-independent apoptosis. By using GAPDH as the control of cytoplasmic protein and Histone H3 as the control of cytoplasmic protein, it was found that CpG 685 can decrease AIF expression in cytoplasmic while increasing AIF expression in the nucleus in BLIN-1 and RS4;11 cells. Columns in the right panel represent means of at least 3 independent experiments; bars represent SD. *p<0.05, **p<0.01.

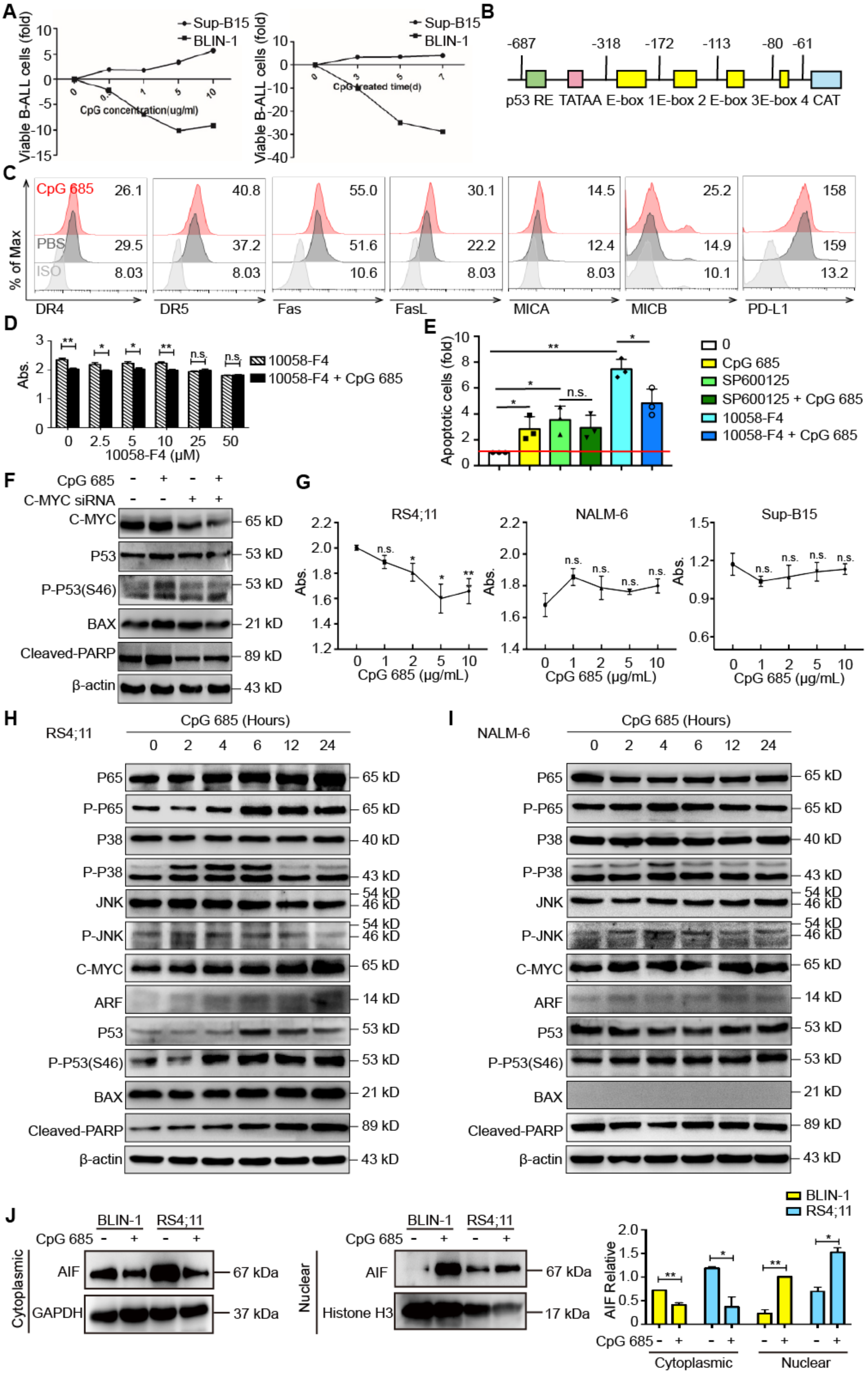
A
Viable B-ALL cells (fold)
Sup-B15
BLIN-1
CpG concentration(ug/ml)
CpG treated time(d)
B
-687
-318
-172
-113
-80
-61
p53 RE
TATAA
E-box 1
E-box 2
E-box 3
E-box 4
CAT
C
CpG 685
PBS
ISO
% of Max
DR4
DR5
Fas
FasL
MICA
MICB
PD-L1
D
Abs.
10058-F4
10058-F4 + CpG 685
10058-F4 (μM)
E
Apoptotic cells (fold)
0
CpG 685
SP600125
SP600125 + CpG 685
10058-F4
10058-F4 + CpG 685
F
CpG 685
C-MYC siRNA
C-MYC
P53
P-P53(S46)
BAX
Cleaved-PARP
β-actin
G
RS4;11
NALM-6
Sup-B15
CpG 685 (μg/mL)
H
RS4;11
CpG 685 (Hours)
P65
P-P65
P38
P-P38
JNK
P-JNK
C-MYC
ARF
P53
P-P53(S46)
BAX
Cleaved-PARP
β-actin
I
NALM-6
CpG 685 (Hours)
J
BLIN-1
RS4;11
CpG 685
Cytoplasmic
Nuclear
AIF
GAPDH
Histone H3
AIF Relative

Additional file 1: Figure S3

(A) Sup-B15 cells are resistant to CpG 685 treatment in vitro. The WST-1 cell viability assay showed kinetic changes in Sup-B15 cell number after 3-day culture in media with different doses of CpG 685. Columns represent means of 5 independent experiments; bars represent SD.

(B) CpG 685 cannot promote Sup-B15 cell apoptosis. Viable Sup-B15 cells cultured in media with different doses of CpG 685 after 3-day culture were examined by annexin-V/7AAD staining. The percentage of apoptotic Sup-B15 cells was calculated.

(C) CpG 685 cannot upregulate the immune regulatory molecules expression on Sup-B15 cells. CD40, CD80, CD86, MHC-I, and MHC-Ⅱ expression were tested by flow cytometry on Sup-B15 with (red-shaded histogram) or without (dark gray-histogram) 5 μg/mL CpG 685 after 24 h culture.

(D) Immune regulatory molecules expression on Sup-B15 cells with CpG 685 (red shaded-histogram) or without CpG 685 (dark-shaded histogram) after 24 h culture. DR4, DR5, Fas, FasL, MICA, MICB, and PD-L1 expression were tested by flow cytometry.

(E) Sup-B15 cells with or without SB203580 pretreatment were cultured in media with or without CpG 685 for 24 h. Western blot results showed that P53 phosphorylation and BAX expression were inhibited by SB203580 pretreatment (left panel). Densitometry of western blots was analyzed with ImageJ and is presented as a mean ± SD at each time point on the right panel.

(F) Although CpG 685 can upregulate the expression levels of BAX in Sup-B15 and BLIN-1 cells, it can active BAX contents as assessed by a conformation-specific antibody directed against the 6A7 epitope in BLIN-1 cells only (left panel). Columns represent means of at least 3 independent experiments; bars represent SD (left panel). Significant differences were accepted at *p<0.05 against the group with no CpG 685 on each cell line.

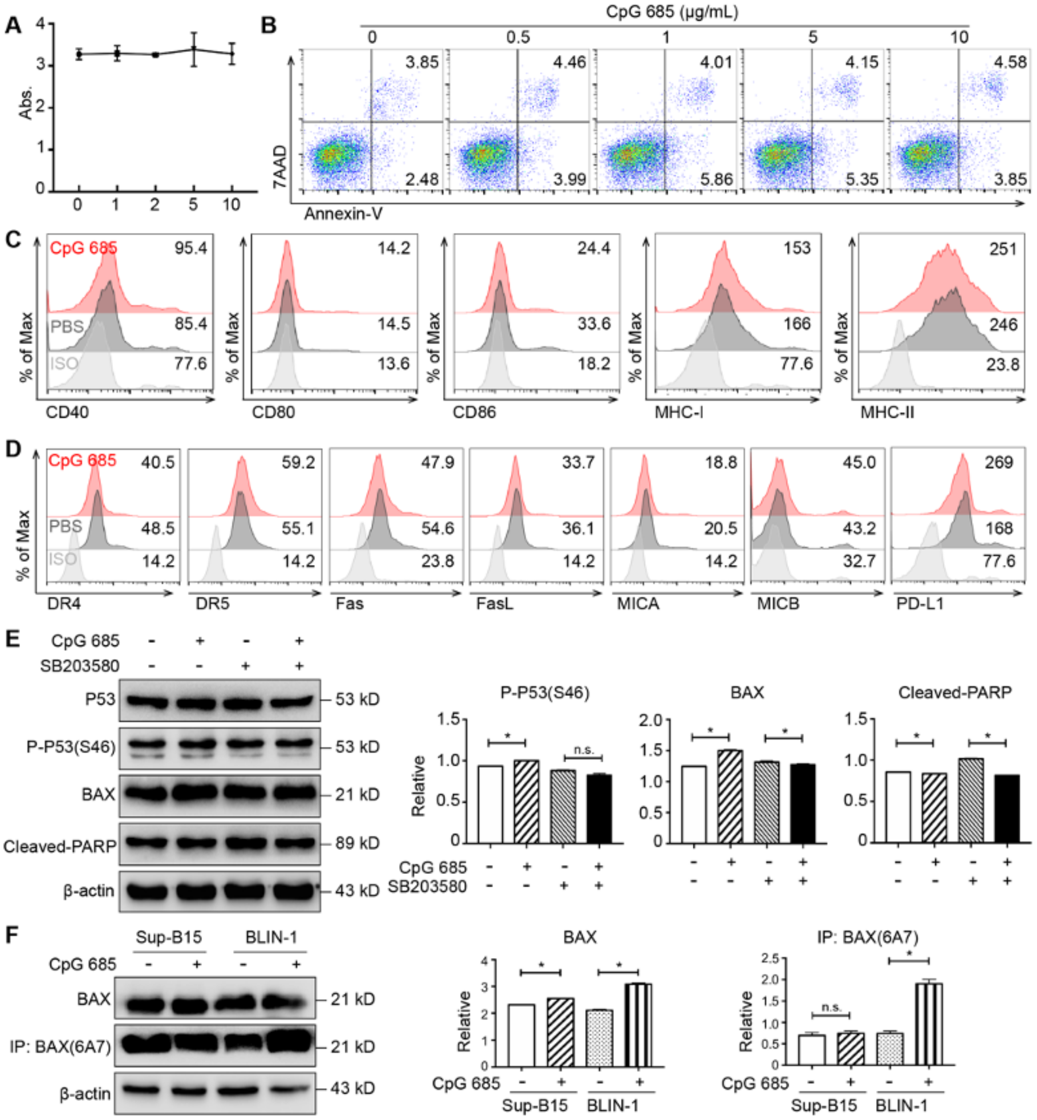

A
Abs.
B
CpG 685 (μg/mL)
7AAD
Annexin-V
C
CpG 685
PBS
ISO
% of Max
CD40
CD80
CD86
MHC-I
MHC-II
D
DR4
DR5
Fas
FasL
MICA
MICB
PD-L1
E
CpG 685
SB203580
P53
P-P53(S46)
BAX
Cleaved-PARP
β-actin
53 kD
21 kD
89 kD
43 kD
Relative
n.s.
F
Sup-B15
BLIN-1
IP: BAX(6A7)

Additional file 1: Figure S4

(A) Sup-B15 cells are imatinib-resistant. WST-1 cell viability assay showed that imatinib cannot inhibit Sup-B15 proliferation obviously.

(B) The imatinib resistance of Sup-B15 is caused by BCR-ABL1-independent AKT phosphorylation. Western blot results showed that imatinib can promote the phosphorylation of AKT at Ser473.

(C) Combined use of CpG 685 and imatinib significantly upregulated CD40, MHC-I, and PD-L1 expression. Immune regulatory molecules expression on Sup-B15 cells without CpG 685 or imatinib (dark gray -histogram) or with the combined use of CpG 685 and imatinib (red-shaded histogram) after 12 h culture.

(D) Effects of CpG 685 and imatinib on the survival of Sup-B15 mice. Imatinib combined with CpG 685 significantly prolonged median survival time compared with PBS group (median survival: 85 days *vs.* 60 days, p=0.0001). The risk of death in the combination group was 0.21 times higher than that in PBS group.

(E) In the two NCG mouse xenograft models (BLIN-1 (left side) and Sup-B15 (right side)), there was no significant change in the body weight of mice injected with CpG compared with those injected with PBS. However, the body weight of the mice in the combined application of CpG and Imatinib group decreased significantly. Columns represent means of 7 independent experiments; bars represent SEM.

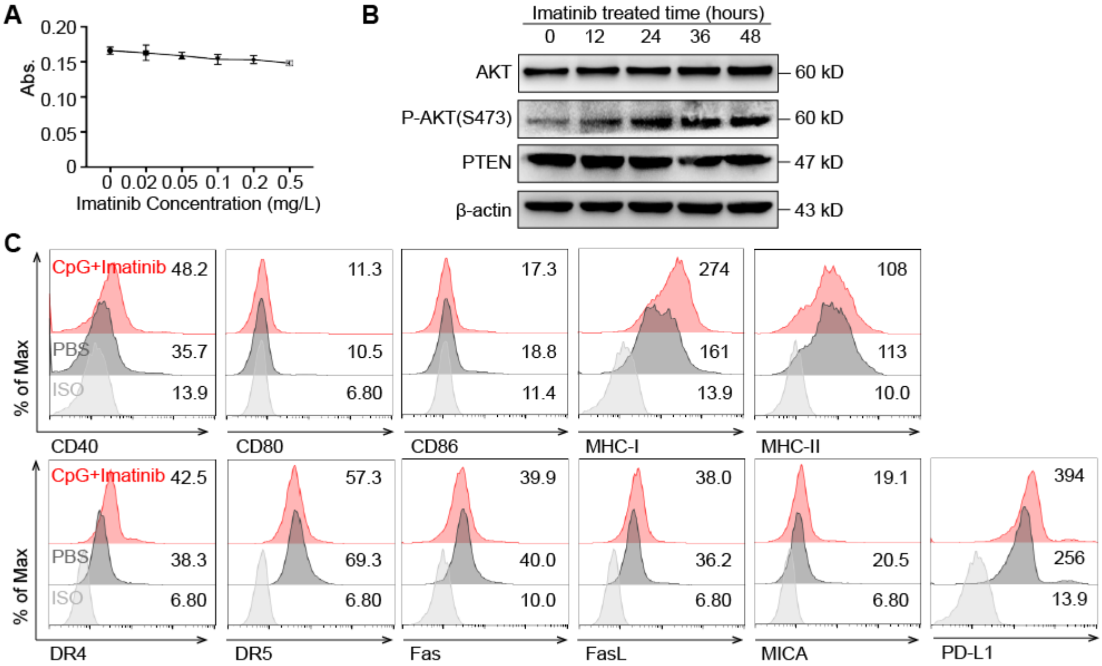

D

Effects of CpG 685 and imatinib on the survival of Sup-B15 mice

Group	N	Median survival (days)	*HR* (log-rank; compared to PBS)	95% *CI* of radio	*P*-value
PBS	7	60 (58 to 67)	-	-	-
CpG	7	74 (70 to 78)	0.210	0.053 to 0.838	0.0001
Imatinib	7	67 (65 to 71)	0.323	0.095 to 1.093	0.0092
Imatinib+CpG	7	85 (82 to 88)	0.210	0.053 to 0.838	0.0001

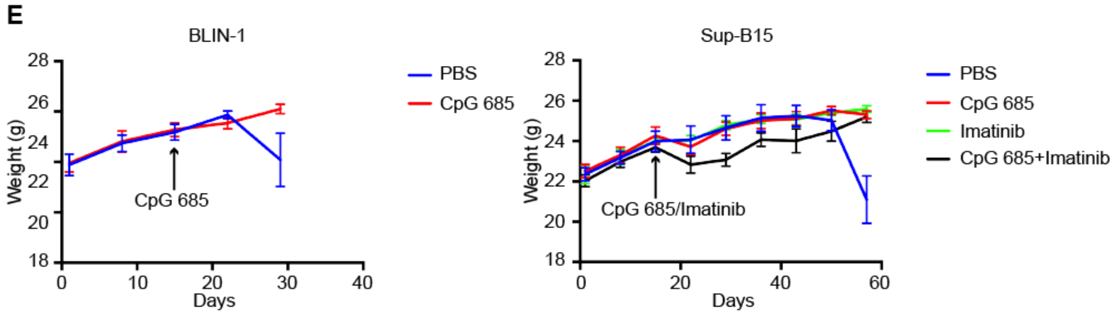

Additional file 1: Figure S5. Patient information of PDX B-ALL model

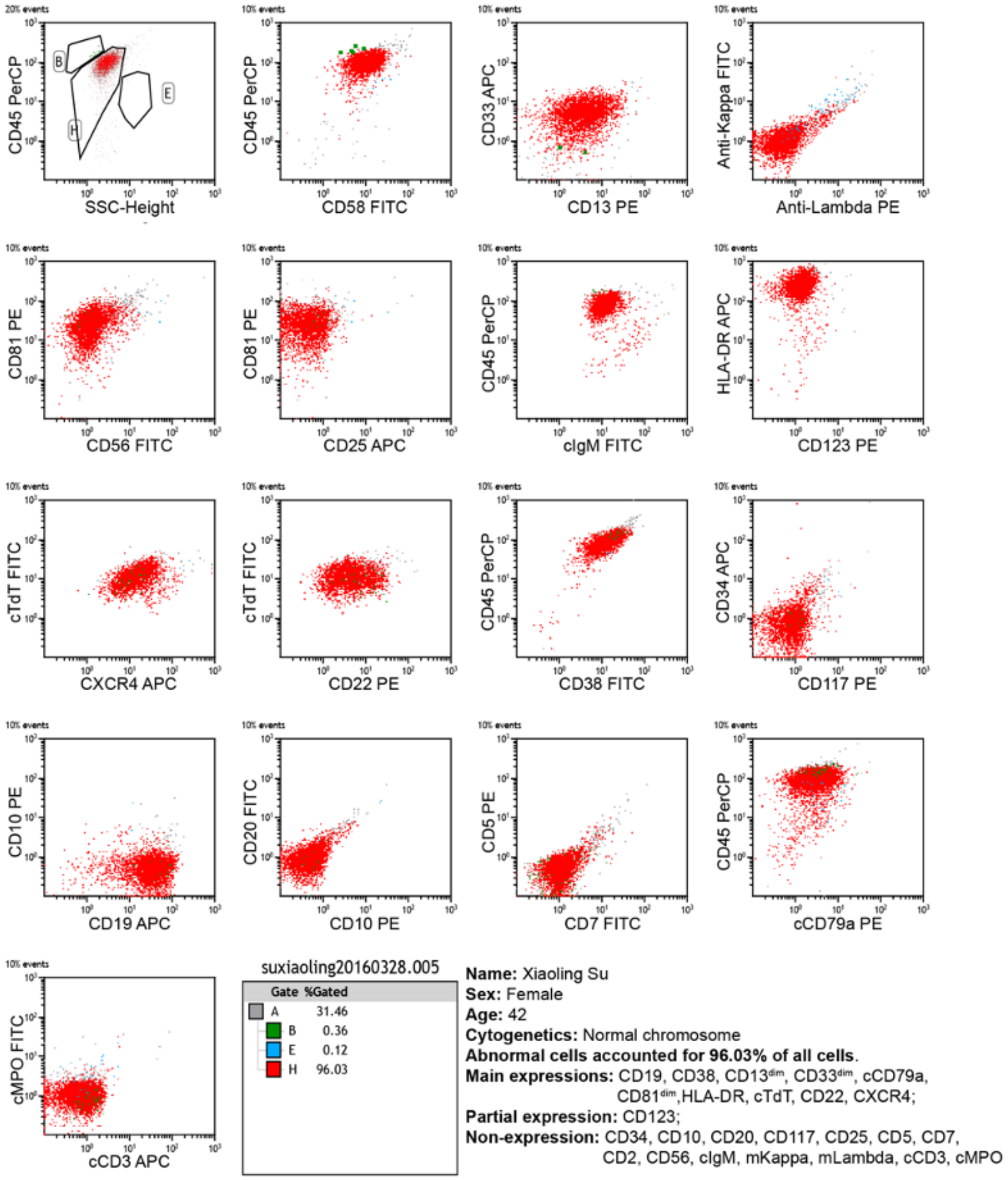

a
